# Acceleration in the DNA methylation age in breast cancer tumours from very young women

**DOI:** 10.1038/s41598-019-51457-6

**Published:** 2019-10-18

**Authors:** Sara S. Oltra, Maria Peña-Chilet, Kirsty Flower, María Teresa Martinez, Elisa Alonso, Octavio Burgues, Ana Lluch, James M. Flanagan, Gloria Ribas

**Affiliations:** 1 Biomedical Research Institute INCLIVA, Hospital Clínico Universitario Valencia, University of Valencia, Valencia, Spain; 2 Department of Surgery and Cancer, Imperial College London, London, UK; 3 Pathology Department, Hospital Clínico Universitario Valencia, University of Valencia, Valencia, Spain; 4 Center for Biomedical Network Research on Cancer (CIBERONC), Valencia, Spain

**Keywords:** Breast cancer, Epigenetics

## Abstract

Breast cancer in very young women (≤35 years; BCVY) presents more aggressive and complex biological features than their older counterparts (BCO). Our aim was to evaluate methylation differences between BCVY and BCO and their DNA epigenetic age. EPIC and 450k Illumina methylation arrays were used in 67 breast cancer tumours, including 32 from BCVY, for methylation study and additionally we analysed their epigenetic age. We identified 2 219 CpG sites differently-methylated in BCVY vs. BCO (FDR < 0.05; β-value difference ± 0.1). The signature showed a general hypomethylation profile with a selective small hypermethylation profile located in open-sea regions in BCVY against BCO and normal tissue. Strikingly, BCVY presented a significant increased epigenetic age-acceleration compared with older women. The affected genes were enriched for pathways in neuronal-system pathways, cell communication, and matrix organisation. Validation in an independent sample highlighted consistent higher expression of *HOXD9*, and *PCDH10* genes in BCVY. Regions implicated in the hypermethylation profile were involved in Notch signalling pathways, the immune system or DNA repair. We further validated *HDAC5* expression in BCVY. We have identified a DNA methylation signature that is specific to BCVY and have shown that epigenetic age-acceleration is increased in BCVY.

## Introduction

Breast cancer (BC) is the most common malignancy in women worldwide^1^. Approximately 6.6% of BCs are diagnosed in women aged 40 or younger, and of all cancers diagnosed in this age group, 40% are BCs; the average risk of developing BC by age 40 is one in 173^2^ and unfortunately, they are not included in mammography screening programmes. BC in very young women (BCVY) (<35 years old) are typically aggressive, in part owing to the over-representation of high-grade, triple-negative tumours (TN), although young age is also an independent negative predictor of cancer-specific survival^3^. Genomic factors, including mutations in tumour suppressor and oncogenes, copy number variation, and epigenetics, are likely implicated in cancer initiation and progression in young women. Different studies have suggested that BCVY presents a different molecular biology to BC in older women and so, should be treated differently^3,4^. Moreover, previous studies from our group^5^ suggest that miRNA expression is different in BCVY and that this may be involved in the increased aggressiveness of tumours in this age group. However, these alterations do not fully explain carcinogenesis and subsequent progression in these young women.

Epigenetic modifications are reported to play an important role in the onset and progression of many diseases. Thus epigenetics, can be used to explain several features of complex diseases, such as the late onset and fluctuation of symptoms^6^. Moreover, epigenetic changes have been well-documented that could change during aging^7^. Several studies on DNA methylation (DNAm) during aging provide evidences that aging is associated with a relaxation of epigenetic control correlated with age related diseases. The functional relevance of age-related epigenetic changes remains largely unknown, possibly with the exception of cancer that is the most important one^8^.

In this study we focused on the differences in genomic methylation between BC tumours from BCVY. We hypothesised that alterations in the BCVY epigenome might contribute to the high aggressiveness of BC in this age group. Understanding the role of the epigenome in younger women may lead to the development of novel epigenetic-based diagnostic strategies, taking the young age of these patients into account.

## Results

### Global hypomethylation in BCVY

**Figure 1:**
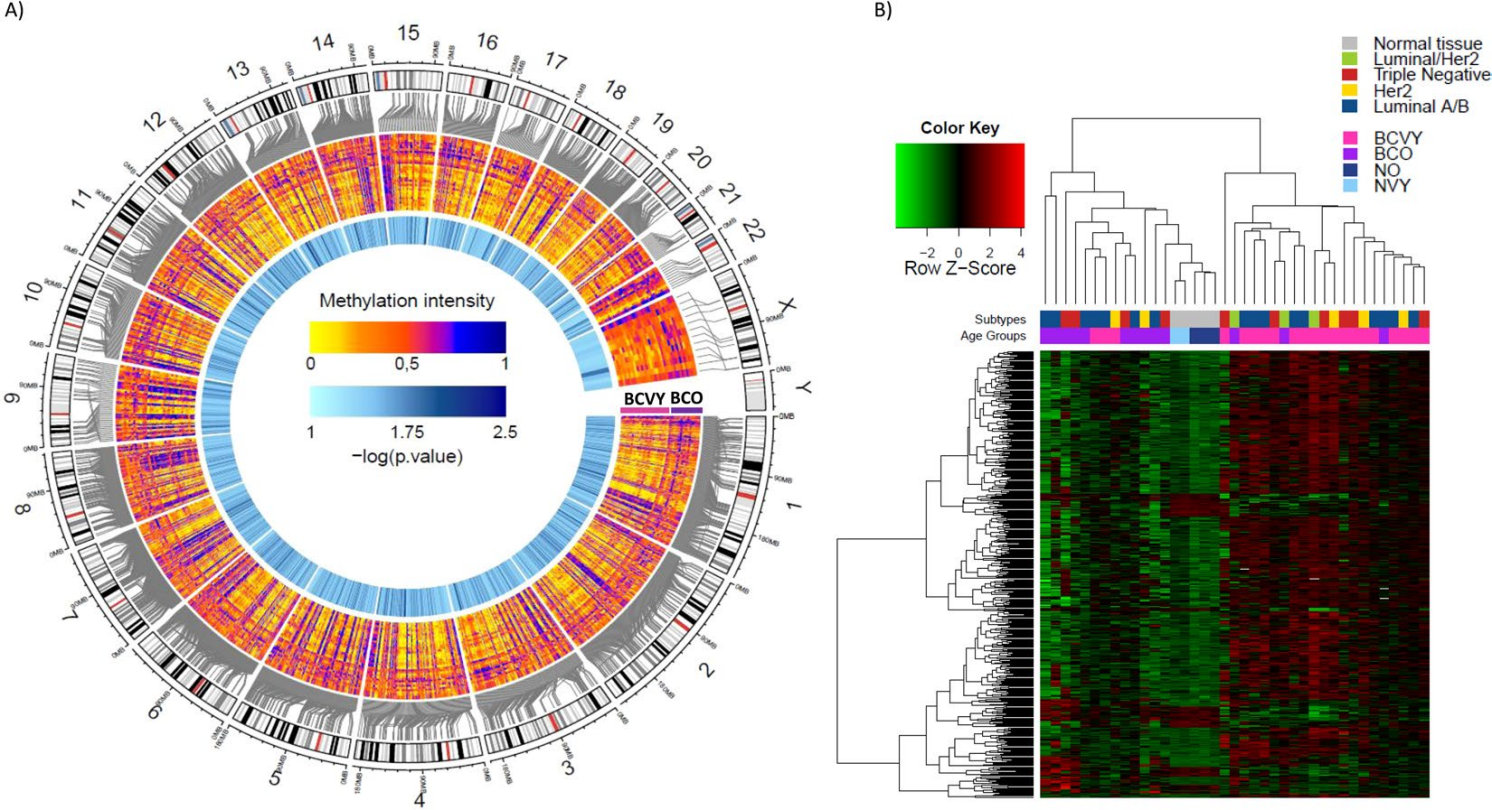
Differential methylation study in breast cancer in very young women (BCVY) vs. their older (BCO) counterparts from metEPICVal samples. (**A**) Circular plot represents DNA methylation levels for significantly differently CpG-methylated probes in BCVY and BCO after removing the methylation differences from normal tissue samples. The average methylation levels in 2219 CpG probes are expressed as β-values (0–1) and are graded from yellow (unmethylated) to blue (methylated). Probabilities are represented as the negative logarithm of the *p-value* graded from light to dark blue (less to more significant); (**B**) Heatmap representing a supervised cluster performed according to a hierarchical clustering methods centred on the median of the methylation levels at the 502 CpG sites distinctive in BCVY compared to BCO and normal tissue samples. Hypermethylated CpG probes (red) and hypomethylated probes (green). Samples studied are represented as BCVY (light pink), BCO samples (purple), normal tissue samples from women younger than 35 years (light blue), and normal samples from women older than 45 years (dark blue). Moreover, subtype bar indicates the molecular subtype for each sample, indicating luminal/Her2 (green), triple negative (red), her (yellow) and luminal A/B subtype (dark blue) and normal tissue is represented in grey colour.

Statistical analysis of the metEPICVal study samples revealed 44 032 CpG sites differently-methylated between BCO vs normal tissue samples and 36 628 CpG for BCVY vs. normal tissues. After removing 18 150 different methylated CpGs that were common to both the control and experimental groups, we analysed the differences in methylation between BCVY and BCO; we identified 2219 CpG sites significantly differentially methylated in BC depending on patient age (Supplementary Fig. S1). There was a general hypomethylation profile in BCVY in which 69% of significant CpG sites were hypomethylated and the remaining 31% were hypermethylated compared to BCO (Fig. 1A).

### Distinctive hypermethylation profile BCVY

We re-analysed methylation differences between BCVY and BCO in the 18 478 BCVY-specific probes. This identified 502 CpG sites that were significantly differently-methylated in BCVY vs. BCO in normal tissue (Supplementary Fig. S1). Of those, 462 CpG sites were also significantly associated with age by GLM analysis (Supplementary Table S1). Only 16% of distinctive BCVY CpG sites were hypomethylated and 84% were hypermethylated. While hierarchical clustering showed two principal sample groups: one consisting of BCVY samples and other including BCO and both normal tissues (NO and NVY), no molecular subtype clusters were found (Fig. 1B). Thus, although BCVY samples are globally hypomethylated, we identified a group of CpG sites with higher methylation, in contrast to the BCO, NVY, and NO samples. We had not an overrepresentation of any subtype among any cancer group (BC *p-value* = 0.72, BCVY *p-value* = 0.098 and BCO *p-value* = 0.472). GLM analysis showed that methylation differences that distinguish BCVY from BCO were specific of age and not related with ER status (*p-value* = 0.21) or molecular subtypes (*p-value* > 0.05) except for Luminal/Her2 which were associated with BCVY methylation differences and this result is in agreement with the major similarity in the methylation profile for BCO-Luminal/Her2 samples with BCVY patients observed in Fig. 1B. However, luminal/her2 sample size is quite limited and no significant conclusions can be drawn. Thus methylation differences observed indicate a BCVY specific methylation signature independent of molecular subtypes and ER status.

### Genomic and functional context of significant CpG sites

**Figure 2:**
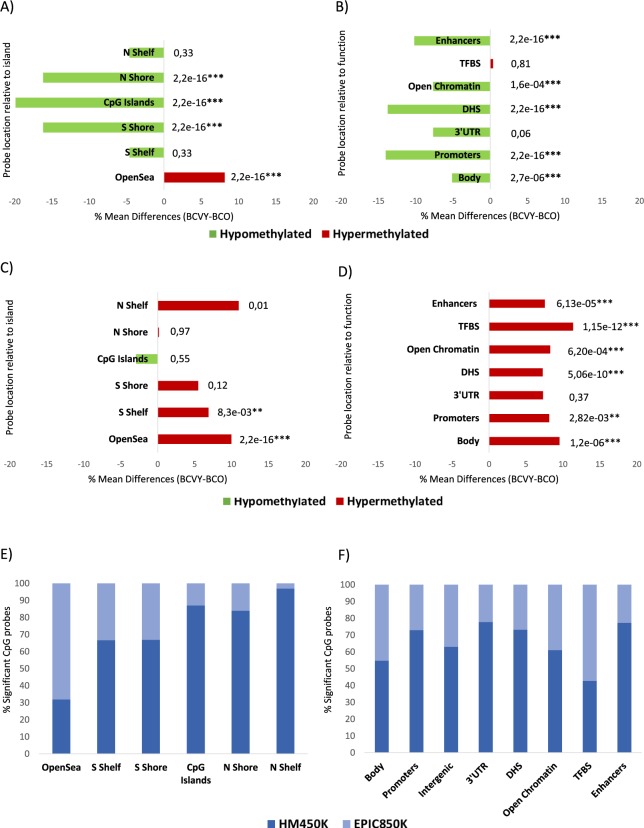
Genomic and functional context of significant CpG sites which are differently methylated in BCVY and BCO from metEPICVal samples analysed by Infinium MethylationEPIC BeadChip, and comparison with HM450K array. Percentage of methylation differences for statistically significant CpG sites from BCVY-BCO comparison according to location of the CpG relative to the island (open-sea, island, N/S shore and N/S shelf) (**A**) and to the UCSC gene region feature category and regulatory elements (**B**); Percentage of methylation differences for distinctive CpG sites in BCVY samples according to the location of the CpG relative to the CpG island (**C**) and to the regulatory elements (**D**). Red bars represent hypermethylation in BCVY and green bars represents hypomethylation; Percentage of significant metEPICVal probes that are in methylation EPIC BeadChip array (EPIC850K) and probes that are common to the EPIC850K and HM450K array (HM450K) according to CpG location (**E**) and functional classification (**F**). Light blue colour represents percentage of significant probes which are in methylation EPIC array and dark blue colour are probes that are as well as in HM450K array. *P ≤ 0.05, **P ≤ 0.01, ***P ≤ 0.001 statistically significant. N/S: north/south; upstream or downstream to the CpG island.

In terms of CpG context, the global methylation differences found in the 2219 CpG probes between BCVY and BCO were significant for CpG islands and the adjacent regions (N/S Shore) and for non-CpG islands (open-sea). However, their specific profiles were different: while CpG islands and near regions (N/S shore) were hypomethylated, open-sea regions were hypermethylated in BCVY samples (Fig. 2A). In terms of region function, the most significant differences were found in proximal promoters (CpG sites located within 200 bp, or 1500 bp upstream of transcription start sites and in 5′UTR) enhancers regions and DNase hypersensitivity sites (*p-value* < 2.2 × 10^−16^). Additionally, we found methylation differences in gene body regions and Open Chromatin. All functional regions for the significant CpG sites analysed were hypomethylated in BCVY (Fig. 2B).

Regarding the hypermethylation profile in BCVY for the 502 CpG probes was localized mainly in open-sea and regions distant from islands (Fig. 2C). Significant CpG probes overlapped in all functional categories included and we were able to find significant methylation differences between BCVY and BCO for all functional categories with the exception of 3′UTR regions that were poorly-represented among significant CpG sites. Interestingly, we found methylation differences in important regulatory genome regions such as enhancers and TFBS (Fig. 2D).

### Methylation in illumina infinium EPIC BeadChip-specific probes

To measure the contribution of EPIC850K to metEPICVal study, we split significant probes into those that were included in both EPIC850K and HM450K arrays from those exclusive for EPIC850K, analysing them according to their functional and genomic distribution. From the 2219 significant probes, 783 were new in EPIC850K array and 1432 were common to both arrays. Interestingly, most of the new and significant EPIC850K probes were hypermethylated in BCVY (65.5%). However, the vast majority of significant hypomethylated probes had already been included in the HM450K. The major contribution of new EPIC850K probes was observed in open-sea and TFBS regions (68% and 57.3%, respectively). About gene context, 45% of significant probes targeting gene bodies were exclusively in EPIC850K array (Fig. 2E,F).

### Accelerated epigenetic aging of BCVY

**Figure 3:**
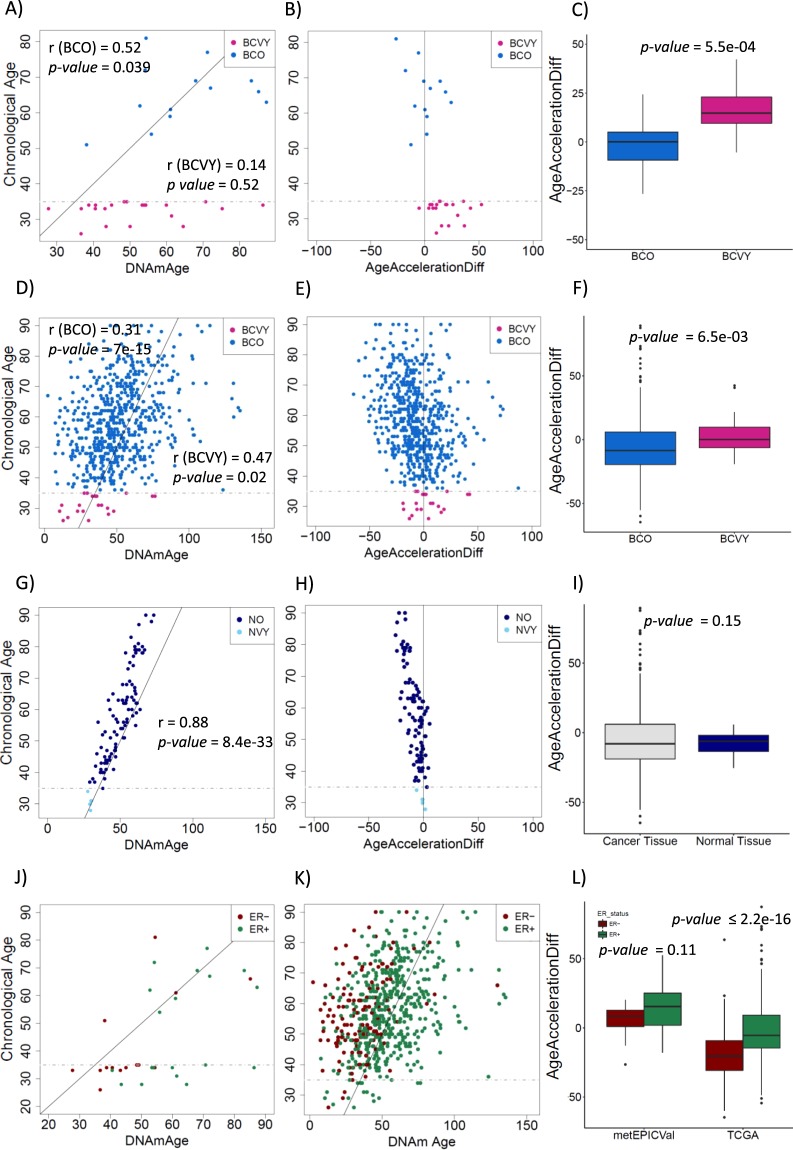
DNA methylation age studies. Results for metEPICVal samples according to: Chronological age versus DNAmAge by age groups (**A**); Age-acceleration differences versus chronological age (**B**); Boxplot for age-acceleration differences between BCO and BCVY (**C**). Analogous results for TCGA samples according to: Chronological age versus DNAmAge by age groups (**D**); Age-acceleration differences versus chronological age (**E**); Boxplot for age-acceleration difference values between BCO and BCVY (**F**). Normal breast tissue results: Chronological age versus DNAmAge by age groups (**G**); Age-acceleration differences versus chronological age (**H**); Comparison of age-acceleration difference values between cancer and normal tissues (**I**). Representation of chronological age versus DNAmAge by oestrogen receptor positive and negative for metEPICVal (**J**) and TCGA samples (**K**); Age-acceleration differences by oestrogen receptor status for metEPICVal and TCGA data sets (**L**). Correlation values (r) for chronological age and DNAmAge by age group are included in plot representation.

We estimated the DNAmAge for metEPICVal samples (Supplementary Table S2). Mean DNAmAge for BCVY was 51.44 years and for BCO was 64.98 years. We observed in metEPICVal sample set a significant correlation between chronological age and DNAmAge for BCO tumours (r = 0.52, *p-value* = 0.039). Although, no significant age correlation was found for BCVY (r = 0.14, *p-value* = 0.52), results shown higher age-acceleration significantly different to BCO (*p-value* = 5.5 × 10^−4^) (Fig. 3A–C). We have reproduced the same analyses to TCGA methylation data detecting also a significant age-acceleration for BCVY (*p-value* = 6.5 × 10^−3^) (Fig. 3D–F). We also examined DNAmAge vs. chronological age in normal samples from TCGA data, identifying an extremely good correlation decreasing in women with advanced age (r = 0.88, *p-value* = 8.4 × 10^−33^) (Fig. 3G,H). Despite homogeneously low age-accelerated values for healthy tissues compared with a more dispersed data in cancer samples, non-significance was reached (Fig. 3I).

Strikingly, we observed an increased age-acceleration in BCVY-ER+ samples for metEPICVal set (*p-value* = 0.001), with a similar trend in the BCVY-TCGA cohort (*p-value* = 0.08). Nevertheless, we found an enrichment of ER+ tumours for BC samples with increased DNAmAge acceleration and we corroborated this association with TCGA (*p-value* < 2.2 × 10^−16^) (Fig. 3J–L). Age-acceleration was not associated with relapse or clinical subtypes. Nonetheless, there was an enrichment of luminal/her2 subtype in BCVY samples with increased age-acceleration values for metEPICVal samples that could not be reproduced in TCGA data. Similarly, all BCVY samples with relapse presented increased age-acceleration compared with BCO samples that suffered a relapse (results not shown). However, there is a limitation in the BCVY sample size in TCGA data and no conclusions can be drawn.

### Validation of methylation differences in combined and TCGA data

We next moved to validate our DNAm findings in BCVY and BCO metEPICVal samples in two 450K data sets (combined data and TCGA). The combined study includes methylation values for 72 samples and 747 samples were included in TCGA data. Considering significant metEPICVal probes present in HM450K (1432 probes) for the global hypomethylation profile in BCVY, 657 were also significant in the combined study (45.8%) (Supplementary Fig. S2a). A minor fraction was also obtained in the TCGA population (116 out of 1254 CpG sites β-values available, 9.25%) (Supplementary Fig. S2b). In both validation cohorts, the vast majority of significant probes were hypomethylated in BCVY (99% in combined data and 95,7% in TCGA data).

Additionally, 35.06% and 25.29% of the total significant probes in the distinctive BCVY methylation profile were also included in HM450K array in combined and TCGA data, respectively. After performing the Wilcoxon rank sum test restricted to significant CpG sites from distinctive hypermethylation signature in BCVY included in HM450K validation sets we observed 15 significant CpG sites of 176 for the combined data and 10 significant CpG sites of 127 for TCGA.

Based on localization and function of the probes, all categories presented significant hypomethylation for BCVY (*p-value* < 2,2 × 10^−16^). Nevertheless, validation studies were missing most of the probes located in enhancer regions, TFBS, Open Chromatin and open-sea regions, exclusive to the EPIC850K array.

### Gene expression TCGA

From the 2219 significant CpG probes from the hypomethylation profile, 573 target genes were both significantly differentially expressed and regulated in BCVY and BCO women. Next, we filtered these significant genes according to their correlation between methylation and gene expression, retaining BCVY-sample overexpressed genes which were regulated by hypomethylated probes as well as repressed genes regulated by hypermethylated probes, to obtain a total of 186 genes with an expression–methylation correlation. Gene expression for genes regulating CpGs sites from BCVY distinctive profile were analysed following the same procedure. We could identify a set of 44 genes differentially expressed in BCVY and regulated by regions differentially methylated.

**Figure 4:**
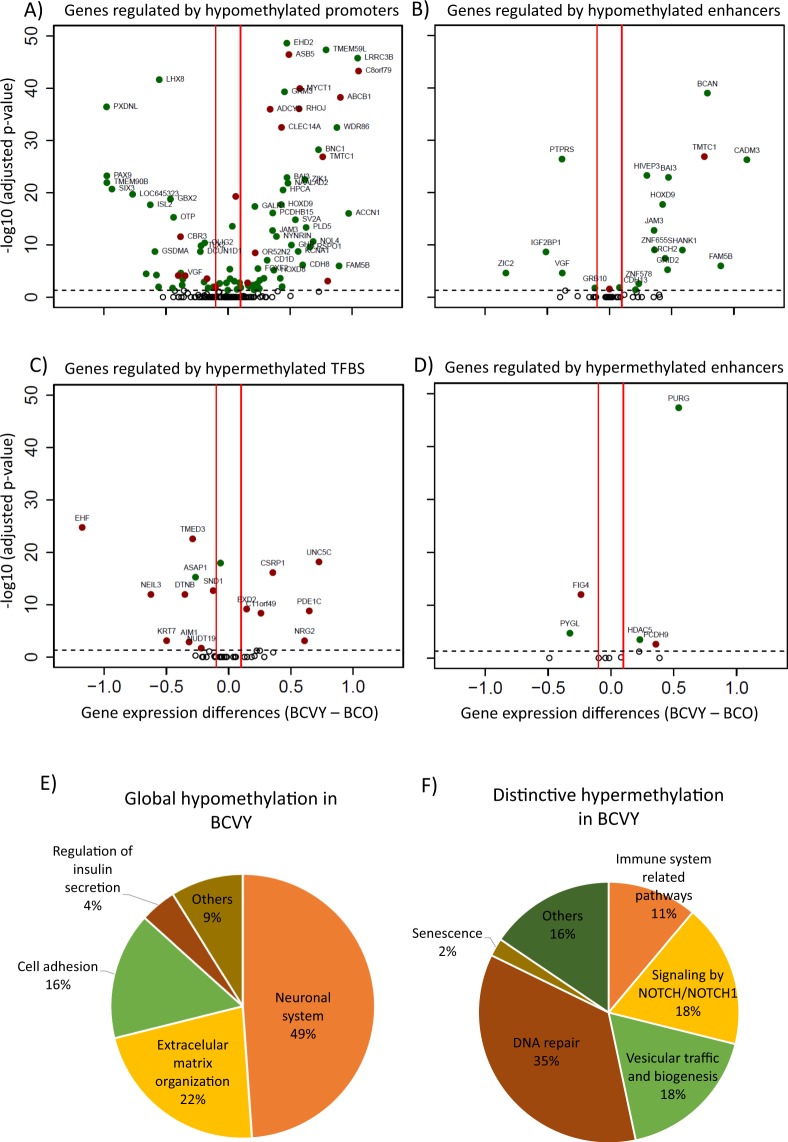
TCGA expression for genes regulated by significantly different methylated CpG sites and pathway enrichment analysis. Genes regulated by differently methylated CpG sites from global BCVY hypomethylation profile found in metEPICVal samples localized in promoters (**A**) and enhancers (**B**). Genes regulated by differently methylated CpG sites from distinctive BCVY hypermethylation signature found in metEPICVal samples localized to TFBS (**C**) and enhancers (**D**); Green dots represent genes regulated by hypomethylated CpG regulatory sites and red dots those regulated by hypermethylated CpG regulatory sites; Percentage of significant enriched signalling pathways associated to genes differently expressed and regulated by global hypomethylated CpG sites in BCVY (**E**) and regulated by distinctive CpG probes generally hypermethylated in BCVY (**F**). EMO: extracellular matrix organization; NOTCH: Signalling by NOTCH/NOTCH1; Cell-Cell: cell communication.

Global hypomethylation was detected in different functional category regions, however, important regulatory elements such as promoter and enhancer sites were highly enriched in the hypomethylation pattern and gene expression for genes affected was evaluated using TCGA data (Fig. 4A,B respectively). Methylation list with probes for promoters and enhancers are included in Supplementary Tables S3 and S4 respectively. TFBS and enhancers regions were significantly represented in the hypermethylated distinctive profile found in BCVY and we analysed expression of genes regulated by them using TCGA data (Fig. 4C,D respectively). Methylation list with probes for enhancers and TFBS are included in Supplementary Tables S5 and S6, respectively.

### Pathway enrichment analysis

Significant genes from the global hypomethylation profile in BCVY were involved in neuronal system pathways related with transmission across synapsis, GABA receptor activation and different neurotransmitter cycles among others. Additionally, 22% of pathways were related with extracellular matrix organization including sulfate/heparin metabolism, proteoglycans metabolism or fibroblasts activation. Pathways related with cell adhesion represented 16% of the significant pathways (Fig. 4E). Complete pathway enrichment is included in Supplementary Table S7. The enrichment pathway analysis for significant genes that characterize exclusively BCVY showed interesting pathways deregulated (Supplementary Table S8). One of the most noteworthy was related with Notch and Notch1 signalling (18%). Immune system pathways represented 11% of total in which Major Histocompatibility Complex I and II were important. Pathways related with DNA repair and vesicular traffic and biogenesis were also highly represented (Fig. 4F).

### Validation step by qRT-PCR

**Figure 5:**
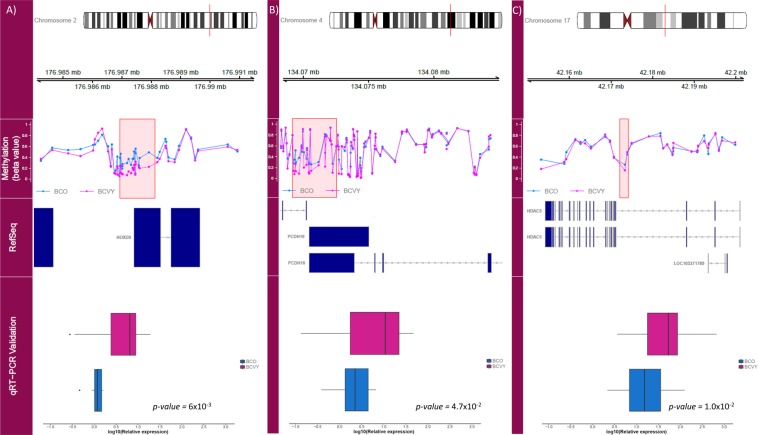
Representation of methylation in the regulatory regions for significantly differently-expressed and validated genes. First track represents the chromosome position of the different methylated region. Methylation track shows the β-values for BCVY (pink) and BCO (blue) methylation. RefSeq track represents genome position of the significant CpG regions obtained. Validation results from qRT-PCR plotted in the qRT-PCR Validation track show gene expression for BCVY (pink) and BCO (blue). (**A**) *HOXD9* methylation profile and validation analysis; (**B**) *PCDH10* methylation profile and validation analysis; (**C**) *HDAC5* methylation profile and validation analysis. Measures of the expression were quantified using a qRT-PCR technique and calculated using the ∆∆Ct method, X axis represents logarithmic transformation of the relative expression. Boxplots represent the sample distribution with the mean for BCO and BCVY patients. All genes were significant (p-value ≤ 0.05) according to the Wilcoxon rank sum test.

In order to validate gene expression obtained for the global hypomethylation, we selected 3 hypomethylated genes (*FOXI2*, *HOXD9* and *PCDH10*). Among them, HOXD9 (*p-value* = 6 × 10^−3^) and PCDH10 (*p-value* = 4.7 × 10^−2^) were significantly upregulated in BCVY previously observed in TCGA gene expression data (Fig. 5A,B).

In addition, for the distinctive hypermethylation profile in BCVY, we selected *APIS3*, *APIB1*, *TIGIT* and *CXCL17* for their involvement in pathways related with immune system, *HDAC5* and *HDAC9* because they were implicated in Notch/Notch1 signalling pathways, *NEIL3* for its involvement in DNA repair, and *EHF*, *PAPSS2*, *PEX*3 and *OTUD3* because of their association with carcinogenesis. Six of them were confirmed to have a differential expression across groups: *APIB1* (*p-value* = 3.7 × 10^−2^), *TIGIT* (*p-value* = 8.1 × 10^−2^), *HDAC5* (*p-value* = 1.0 × 10^−2^), *HDAC9* (*p-value* = 3.7 × 10^−2^), *NEIL3* (*p-value* < 1 × 10^−4^) and *PAPSS2* (*p-value* = 1 × 10^−3^). Despite significant differences in gene expression, only *HDAC5* expression was consistently upregulated in both the qRT-PCR validation and TCGA data (Fig. 5C). As expected, *EHF*, *CXCL17* and *AP1S3* were downregulated in BCVY, however the difference did not reach statistical significance. The mean expression values for each gene obtained in the validation step are included in Supplementary Table S9. The putative functional implications of the genes whose expression was consistent with our TCGA study are summarised in Table 1.

**Table 1:** Published information about validated genes and implication in cancer.

Gene	*p-value*	Function	Published papers
*HDAC5*	0.01**	Histone deacetylases (HDACs) constitute a family of enzymes that play important roles in the epigenetic regulation of gene expression and contribute to the growth, differentiation and apoptosis of cancer cells. *HDAC5* was extensively expressed in human BC tissues, and high *HDAC5* expression was associated with an inferior prognosis. *HDAC5* enhances Notch 1 expression at both the mRNA and the protein level in glioma cell lines. *HDAC5* promotes glioma cell proliferation, suggesting that this effect involves the upregulation of Notch 1.	^28, 33^
*HOXD9*	0.005**	Hox gene expression is regulated by epigenetic control. CpG islands in Hox promotor genes are commonly methylated. Gene expression alteration could be important in oncogenesis and tumour suppression. *HOXD9* activation is a negative prognostic marker in glioblastoma, hepatocellular carcinoma, and cervical cancer.	^37^
*PCDH10*	0.048*	Protocadherin 10 is a tumour suppressor gene, and is frequently inactivated epigenetically in multiple carcinomas: pancreatic cancer, gastric cancer, prostate cancer, breast cancer, and other carcinomas.	^38^
*EHF*	0.248	*EHF* overexpression has been related with activation of HER family and poor survival in gastric cancer. The *EHF* gene has been characterised as a breast, prostate, and lung tumour suppressor gene. Loss of *ESE3/EHF* induces epithelial mesenchymal transition, stem cell-like features, and tumour-initiating and metastatic properties in prostate epithelial cells.	^39, 40^
*CXCL17*	0.502	Chemokine ligand 17 levels are upregulated in the early intraepithelial stages of human pancreatic carcinogenesis and are involves in increasing anti-tumour immune response. For hepatocellular carcinoma *CXCL17* is mainly produced by tumour-infiltrating neutrophils and can be used as an independent indicator of poor prognosis.	^41^
*AP1S3*	0.740	Adaptor protein complex 1 (*AP-1*) is an evolutionary conserved heterotetramer that promotes vesicular trafficking between the trans-Golgi network and endosomes. *AP1S3* silencing disrupts the endosomal translocation of the innate pattern-recognition receptor *TLR-3* (Toll-like receptor 3) and results in marked inhibition of downstream signalling.	^42^

Table includes statistically significant genes that were validated by qRT-PCR which expression was correlated with gene expression from TCGA analysis. Moreover, we included non-significant genes whose expression were consistent with TCGA study results.

*Refers to significant *p-values* analyzed by Wilcoxon rank sum test: *P ≤ 0.05, **P ≤ 0.01, ***P ≤ 0.001.

## Discussion

The overall pattern shows a global hypomethylation signature in BCVY which is irrelevant of probe location. Significant hypomethylation was detected in CpG Islands and adjacent regions located mainly in promoters and enhancer regulatory regions. Several epidemiological case-control studies have reported global hypomethylation in peripheral blood of cancer patients, suggesting systematic effect of hypomethylation on disease predisposition^9,10^. However, our results reported that global hypomethylation is presumably more important in BCVY. In addition, we have identified a distinctive signature of hypermethylation in 502 CpG sites characteristic of BCVY tumours, localized mainly in regions around CpG islands and open-sea, as well as, overlapping in important regulatory expression sites such as enhancers and TFBS. Previous works revealed distinctive methylation patterns among BC subtypes, with hypermethylation for luminal B and hypomethylation for TN^11,12^. TN-BC subtype is more frequent in young women^13,14^ and although in our sample set we have several TN-BC, these are not significantly overrepresented in BCVY samples and GLM analysis demonstrate that methylation differences observed are characteristic of BCVY and independent of molecular subtypes and ER status.

According to the literature, with few exceptions, mammalian aging is more commonly associated with CpG hypomethylation patterns, especially at repetitive DNA sequences^15^. Age-related DNAm changes are also more prominent in CpG islands^16^. Thus, while general and localised DNA hypomethylation is detected during aging, hypermethylation would simultaneously occur at specific CpG sites with the latter presumably repressing the expression of particular genes^17–19^. Therefore, we would expect higher hypomethylation in CpG island regions for older women. On the contrary, our results show stronger CpG island hypomethylation in BCVY samples. Several known CpG sites that are predictive of age have been identified in different studies^16,18,19^ and therefore, these probes have been excluded from our analysis to avoid that the results were confounded by age related methylation changes.

The global hypomethylation identified in the BCVY group, leads us to infer a higher gene deregulation in addition to a greater genomic instability, that could justify the aggressiveness of these tumours^15^. Additionally, we have detected noticeable discrepancies between chronological and DNAmAge in BCVY tumours, which results in a significant increased age-acceleration compared with BCO in both datasets analysed.

Previous studies suggest that epigenetic age-acceleration might also be an indicator for a more aggressive course of tumour disease^20,21^. This age-acceleration has been seen in a previous study in breast tissue from healthy females respect its paired blood sample^22^. However, normal breast tissue from TCGA shown a strong correlation between chronological age and DNAmAge. This correlation is better than the one obtained for cancer tissues, suggesting that acceleration observed in BCVY is disease specific and not tissue related.

Furthermore, we observed a higher age-acceleration for ER+ tumours in both data sets. These results support previous observations from Horvath and colleagues^18,19^. Although we should expect higher acceleration in older women, because their major proportion of ER+ tumours and negative acceleration values for BCVY because aggregates major proportion of TN subtypes^18,19^, our results strongly indicate a higher age-acceleration in BCVY. Additionally, we found that BCVY metEPICVal samples that were ER+ presented a significant higher epigenetic age, a trend seen also in the BCVY samples from TCGA dataset.

BC risk is linked with endogenous hormone exposure which is reduced in the post-menopausal status. Thus, we hypothesized that the acceleration found in BCVY may be partially influenced by ER status, but another factors such as hormonal levels could be involved in the increased DNAmAge and contributing with their poor outcome.

We could reproduce global hypomethylation found in BCVY, both in combined study and TCGA data sets with 99% and 95,7% significant probes respectively. We could not detect the distinctive hypermethylated profile for BCVY in any of the two validation populations, due to the low proportion of open-sea probes in the HM450K array. Despite that, CpG shores, shelves and open-sea regions has been associated with different carcinomas and, reprogramming-specific differentially methylated regions, with mechanistic relevance for the expression of associated genes^23,24^. Our study suggests that open-sea regions may play an important role in gene regulation in different cancer types and specifically in BCVY, but because these regions are underrepresented in HM450K studies, further research will be required to test this hypothesis.

Furthermore, we showed that global hypomethylation in BCVY was significantly overrepresented in genes involved in neuronal-system processes, as well as, in extracellular matrix organisation and cell communication. In agreement with these results, previous articles have highlighted the importance of neural elements in the cancer microenvironment in which promotes cell growth. However, the mechanisms mediating neuronal influences on cancer growth and progression are likely incompletely understood^25^.

Gene expression analysis of TCGA data identified 186 genes whose expression correlated with the global hypomethylation signature found in BCVY. As expected, two of three genes included in our validation analysis in a different sample set, *HOXD9* and *PCDH10*, were also overexpressed in BCVY. Both, promoter and enhancer CpG sites of HOXD9 were hypomethylated. Previous works showed that silencing *HOXD9* in liver cancer inhibited epithelial–mesenchymal transition, migration, and invasion *in vitro* and decreased tumorigenic and metastatic capacities *in vivo*^26^. This phenomenon is essential for tumour cells dissemination and metastasis and fits with the major invasive ability of BCVY tumours.

While Protocadherin 10 (*PCDH10)* overexpression has been related to inhibition of invasion and metastasis^27^. Our results showed hypomethylation in a *PCDH10* gene-regulation region and correlates with higher expression of this gene in BCVY, as also validated by qRT-PCR.

Further, we found that hypermethylation in BCVY modulates characteristic pathways related with immune system, DNA repair, vesicular trafficking and biogenesis as well as with Notch/Notch1 signalling, and probably has a role in the aggressive pathophysiology of the disease. Of the 11 genes selected from the distinctive BCVY for validation step, only *HDAC5* showed results coherent with TCGA gene expression. Despite the hypermethylated profile, *HDAC5* CpG regulatory regions, and specially enhancer sites, were hypomethylated and was coincident with overexpression validated in BCVY. *HDAC5* is a histone deacetylase that enhances Notch1 expression in glioma cell lines promoting cell proliferation^28^. Notch signalling can be either oncogenic or tumour suppressive in distinct human cancers, or may even adopt both roles in the same tumour type^29^. Interestingly, previous work found expression signatures related to Notch signalling pathways in BC patients younger than 45 years old^30^. As for *HDAC5*, high expression has been correlated with poorer prognosis in patients with BC or pancreatic neuroendocrine tumours and has been attributed with oncogenic effects^31,32^. Moreover, *in vitro* studies suggest that the HDAC5 inhibitor, LMK-235, could be a novel therapeutic strategy for treating BC^33^.

Our observations suggest that the methylation patter seen in BCVY differs to that observed in BCO. BCVY presents a global hypomethylation profile that can be validated with TCGA data, and a specific hypermethylation signature seen in metEPICVal samples, mainly present in open-sea regions, that could play an important role in the biological complexity of BCVY tumours. Hypomethylation signature found in BCVY may be contributing to the increased DNAm age-acceleration detected. We propose that BCVY aggressiveness could be potentiated by genome instability triggered by global hypomethylation found. Moreover, hormonal factors affecting young women could be responsible of the accelerated DNAmAge and may be associated with the poor prognosis of BCVY, in which hormone exposure are high and more important than in BCO. Therefore, the differences observed in BCVY in our work and others, strengthen the hypothesis that BC arising in young women has a potentially unique, aggressive and complex biological features and more efforts should be done to reach a more personalized treatment.

## Materials and Methods

### Sample data sets for DNA methylation assays

#### Illumina infinium methylationEPIC BeadChip samples from hospital clínico universitario of valencia (metEPICVal)

All the samples included in the Illumina Infinium MethylationEPIC BeadChip (metEPICVal samples) were archived formalin fixed paraffin-embedded (FFPE) BC tissues stored at the *Hospital Clínico Universitario Valencia*, Spain. The clinical characteristics of the patients are shown in Table 2. This study was approved by the *Comité Ético de Investigación Clínica del Hospital Clínico Universitario de Valencia* (CEIC-HCUV) and an informed consent was obtained from all participants and/or their legal guardians. All experiments were performed in accordance with relevant guidelines and regulations.

**Table 2:** Clinical tumour characteristics of samples from the metEPICVal and the qRT-PCR validation sample set.

	Discovery set n = 34		Validation set n = 40	
	BCVY (n = 21)	BCO (n = 13)	BCVY (n = 27)	BCO (n = 13)
**Age mean** (**SD**)	32.5 (2.7)	65.5 (8.5)	31.8 (3.2)	68.2 (7.3)
**Histological subtype** (**%**)				
Luminal A	2 (9.5)	2 (15.4)	3 (11.1)	4 (30.8)
Luminal B	8 (38.1)	4 (30.8)	11 (40.7)	5 (38.5)
TN	6 (28.8)	4 (30.8)	6 (22.2)	1 (7.7)
Luminal/HER2	1 (4.7)	2 (15.4)	1 (3.7)	2 (15.4)
HER2	4 (19.0)	1 (7.6)	6 (22.2)	1 (7.7)
**ER status** (**%**)				
ER positive	11 (52.4)	9 (69.2)	15 (55.5)	10 (76.9)
ER negative	10 (47.62)	4 (30.8)	10 (37.0)	3 (23.1)
**Relapse** (**%**)	4 (19.05)	1 (7.69)	7 (25.9)	0

Abbreviation: SD: standard deviation; TN: triple negative subtype; ER: oestrogen receptor.

#### Infinium HumanMethylation450 BeadChip samples

We included methylation data from a previous study^34^ which used Infinium HumanMethylation450 BeadChip (IC-HM450K) (GSE72277). We retaining 11 samples from women aged younger than 35 years and 22 from women older than 45 years.

#### The cancer genome atlas data

We accessed methylation and gene expression data from The Cancer Genome Atlas (TCGA). Methylation study was performed by HM450K array and includes data for 485 577 probes in 720 BCO and 27 BCVY samples. Gene expression by RNAseq (IlluminaHiSeq) includes data for 20 530 genes in 1102 BC samples (35 samples from BCVY and 924 from BCO).

### DNA extraction and methylation measurements

The metEPICVal samples were extracted using QIAamp DNA FFPE Tissue Kit (Qiagen, Hilden, Germany). DNA samples were quantified (Quant-iT PicoGreen dsDNA Assay, Life Technologies, CA, USA), and assessed for purity by NanoDrop (Thermo Scientific, MA, USA); 500 ng of FFPE DNA were bisulphite converted using the EZ-96 DNA Methylation-Gold™ kit (Zymo Research Corp., CA, USA) and restored following manufacturer’s instructions. Next, DNA was hybridised to the Illumina Infinium MethylationEPIC BeadChip array (EPIC850K). Raw and normalized microarray data will be available from Gene Expression Omnibus (GEO) (GSE100850). Post-array sample quality control was implemented removing bad quality samples and β-values calculation were performed using *minfi* R package^35^. Single nucleotide polymorphism and cross reactive probes [36] were removed. After pre-processing, we analysed 793 483 CpG sites in 21 samples from BCVY,13 from BCO, 2 NVY and 3 from NO.

### Statistical analysis

The methylation differences were assessed using Wilcoxon rank sum test and generalized linear model (GLM), *p-values* were adjusted by Benjamini-Hochberg procedure to generate a false discovery rate (FDR). We considered significant CpG sites those with both adjusted FDR *p-value* ≤ 0.05 and a minimum change of ±0.1 in β-values. Flow-diagram of the analysis is shown in Supplementary Fig. S1. First, we analysed methylation differences between normal tissue samples (NVY and NO) and both BC data sets (BCO and BCVY) respectively. From significant CpG sites we removed those probes common to both cancer sets. Finally, we analysed only those probes differentially methylated in BCVY and compared the methylation status in BCO. In this case an *p-value* of 0.01 was set. Chi-squared test was used to check differences in the proportion of molecular subtypes and ER status between BC age groups. Additionally, we performed a GLM analysis to assess whether specific methylation differences observed between BCVY and BCO were independent of molecular subtype and ER status.

As previously described SS Oltra et al 2018^36^ Illumina probes were classified into different according to their function (promoter, gene body, TSS or UTRs), relation to the CpG context (N/S shore, N/S shelf and island), enhancer regions provided by ENCODE and FANTOM5 projects and ENCODE regulatory elements (CpG sites in transcription factor biding sites [TFBS], open chromatin regions and DNase hypersensitive regions). Methylation differences by categories were assessed by Welch’s t-test, *p-value* of 0.001 was set.

### Estimation of DNA methylation age

DNAm age (DNAmAge) was calculated for metEPICVal samples. To this end, we used a multivariable linear model based on the DNAm levels of 353 CpGs described by Horvath^18,19^. We calculated both age-acceleration difference and age-acceleration residual^18^. Differences in age-acceleration between BCVY and BCO were analysed by Wilcoxon rank sum test. We used TCGA methylation data for validation study of DNAmAge.

### Validation study of methylation with combined cohort and TCGA data

We performed a combined study using methylation data from IC-HM450K and metEPICVal studies. Retaining methylation levels for 405 068 probes present in both studies in a total of 72 samples (35 BCO samples, 32 from BCVY, 3 from NO and 2 from NVY). TCGA methylation data includes β-values for 485 577 probes in 720 BCO and 27 BCVY samples.

### The cancer genome atlas gene expression

Gene expression regulated by the CpGs sites differentially methylated in BCVY was analysed using RNAseq expression data from TCGA for BC. RNAseq expression study was done with 50 permutations, and samples were randomly selected and balanced by subtype. We used *metap* R package to combine *p-values* for each gene in each study; *p-values* were adjusted by Benjamini-Hochberg FDR procedure; genes with FDR < 0.01 and gene expression differences ±0.1 were considered statistically significant (Supplementary Fig. S1).

### Pathway enrichment analysis

Enrichr (http://amp.pharm.mssm.edu/Enrichr), was used to gain insight into the pathways and biological processes. This software performs an enrichment analysis of multiple genes using different gene-set libraries such as Reactome, Gene Ontology or KEGG. Pathways showing a *p-value* ≤ 0.05 were considered significantly enriched.

### Gene expression validation by quantitative real time-PCR

Gene validation was performed in a different sample set composed of 27 samples from BCVY and 13 from BCO using Quantitative real time-PCR (qRT-PCR) (clinical characteristics in Table 2). RNA from selected samples was isolated using RecoverAll Total Nucleic Acid Isolation Kit (Applied Biosystems by Life Technologies, Carlsbad, California, USA). RNA concentration was measured using a NanoDrop ND 2000 UV–vis Spectrophotometer (Thermo Fisher Scientific Inc., Wilmington DE, USA). We used 100ng of total RNA for reverse transcription-qPCR in order to obtain cDNA using the High Capacity cDNA transcription Kit (Applied Biosystems by Life Technologies, Carlsbad, California, USA). TaqMan Gene Expression Assays (Applied Biosystems by Life Technologies, Carlsbad, California, USA) was used for qRT-PCR for selected genes. Normalisation was done with GAPDH expression. FOXI2 gene probe failed in most of the samples tested and was excluded from this study. We use the Thermofisher Scientific software QuantiStudio Desing and Analysis software (v1.4). Relative expression was calculated by using the comparative Ct method and obtaining the fold-change value (ΔΔCt). The Wilcoxon rank sum test was used for non-parametric samples, *p-value* ≤ 0.05 were considered statistically significant.

## Supplementary information

Supplementary Tables

Supplementary Figures
