# Supplementary material for: Acceleration in the DNA methylation age in breast cancer tumours from very young women
Source: Sci Rep. 2019 Oct 18;9:14991. doi: 10.1038/s41598-019-51457-6 (PMC6800453; doi:10.1038/s41598-019-51457-6)
Supplement: Supplementary file 1 — Supplementary Tables [file 41598_2019_51457_MOESM1_ESM.pdf]

Acceleration in the DNA methylation age in breast cancer tumours from very young women

Sara S. Oltra[1], Maria Peña-Chilet[1], Kirsty Flower2, María Teresa Martinez[1], Elisa Alonso[3], Octavio Burgues[3], Ana Lluch[1¥], James M. Flanagan[2], and Gloria Ribas[1¥]*

Supplementary table S1. **Statistical studies for distinctive hypermethylated CpGs in BCVY from metEPICVal study**. Results for Wilcoxon rank sum test and generalized linear model by age. Mean methylation columns include mean β- values for each significant probe for BCVY and BCO. Table indicates p-values obtained for methylation differences between BCVY and BCO by Wilcoxon rank sum test and generalized linear model by age. Gene/s regulated by each probe are in Gene region column.

Supplementary table S2. **DNA methylation age results for metEPICVal samples.**

Supplementary table S3. Significant CpG probes localized in promotor regions from hypomethylation global profile in BCVY from metEPICVal study. Table include differences in methylation and gene expression between BCVY vs BCO. P-values and adjusted p-value columns have been obtained from TCGA gene expression meta-analysis.

Supplementary table S4. Significant CpG probes localized in enhancer regions from hypomethylation global profile in BCVY from metEPICVal study. Table include differences in methylation and gene expression between BCVY vs BCO. P-values and adjusted p-value columns have been obtained from TCGA gene expression meta-analysis.

Supplementary table S5. Significant CpG probes localized in enhancer regions from hypermethylation distinctive signature in BCVY from metEPICVal study. Table include differences in methylation and gene expression between BCVY vs BCO. P-values and adjusted p-value columns have been obtained from TCGA gene expression meta-analysis.

Supplementary table S6. Significant CpG probes localized in TFBS regions from hypermethylation distinctive signature in BCVY from metEPICVal study. Table include differences in methylation and gene expression between BCVY vs BCO. P-values and adjusted p-value columns have been obtained from TCGA gene expression meta-analysis.

Supplementary table S7. Pathway enrichment results for genes regulated by the CpG probes globally hypomethylated in BCVY. Enrichment was performed in enrichr tool and pathways obtained were provided by Reactome database. All pathways presented a $p < 0.05$ and were classified into different general functional categories.

Supplementary table S8. Pathway enrichment results for genes regulated by CpG probes distinctively hypermethylated in BCVY. Enrichment was performed in enrichr tool and pathways obtained were provided by Reactome database. All pathways presented a *p-value* ≤ 0.05 and were classified into different general functional categories.

Supplementary table S9. Results of qRT-PCR validation analysis. BCVY and BCO mean columns represent mean expression values obtained for each gene. Mean differences column indicates differences between BCVY mean minus BCO mean, positive values are for genes overexpressed in BCVY and negative for genes repressed in BCVY comparing with BCO. The p-values included are from Wilcoxon rank sum test analysis.

Supplementary Table S1. Statistical studies for distinctive hypermethylated CpGs in BCVY from metEPICVal study.

	Mean methylation BCVY	Mean methylation BCO	Wilcoxon rank sum test BCVY vs BCO *p value*	Generalized linear model ~ age BCVY vs BCO *p value*	Gene region
cg00049892	0,54	0,42	4,61E-02	6,14E-02	ENPP1
cg03340334	0,42	0,54	2,18E-02	3,33E-02	MIR1268A
cg19844232	0,62	0,74	1,04E-02	4,31E-02	ASAP1
cg23093939	0,36	0,48	2,65E-02	4,34E-02	
cg16731083	0,43	0,57	2,41E-02	3,65E-02	
cg12493813	0,67	0,54	1,44E-02	2,23E-02	CCDC170
cg08048895	0,60	0,49	2,18E-02	6,10E-02	AUTS2
cg15564444	0,45	0,57	2,65E-02	5,65E-02	IGSF21
cg20956407	0,71	0,58	3,52E-02	2,70E-02	NDST1
cg04903269	0,59	0,72	4,22E-02	5,57E-02	CELF6
cg06573181	0,63	0,52	2,65E-02	2,96E-02	KCNQ5
cg07215395	0,69	0,56	1,04E-02	2,95E-02	CDO1
cg10006933	0,21	0,37	3,86E-02	3,83E-02	BRINP3
cg13284574	0,46	0,58	2,41E-02	3,60E-02	ESPN
cg25563198	0,51	0,40	3,21E-02	7,62E-02	FKBP5; LOC285847
cg14155265	0,64	0,51	1,97E-02	2,65E-02	AK4
cg15988937	0,73	0,62	4,61E-02	4,47E-02	
cg10165543	0,38	0,50	2,65E-02	4,39E-02	KALRN
cg22514173	0,64	0,76	1,78E-02	3,93E-02	
cg16716542	0,64	0,51	1,60E-02	2,54E-02	CDC42BPB
cg22006449	0,77	0,65	2,65E-02	5,00E-02	PPP2R1B
cg12426652	0,47	0,60	4,22E-02	5,57E-02	CLDN14
cg08206482	0,36	0,52	4,02E-03	1,05E-02	ITGAD
cg25093492	0,47	0,34	2,92E-02	4,56E-02	PLCH1
cg04478905	0,54	0,64	4,22E-02	7,01E-02	CLDN14
cg17367688	0,72	0,61	3,52E-02	5,37E-02	
cg07255909	0,63	0,49	1,16E-02	1,90E-02	
cg07070848	0,83	0,72	1,44E-02	3,83E-02	PPP3CA
cg22652022	0,55	0,42	3,52E-02	5,31E-02	EXOC4
cg22542373	0,40	0,51	9,30E-03	2,24E-02	
cg24724990	0,71	0,60	1,97E-02	5,62E-02	TMEM123
cg12033458	0,75	0,60	2,39E-03	2,33E-02	UNC5C
cg15863598	0,72	0,59	9,30E-03	1,64E-02	MYST4
cg10537261	0,46	0,36	9,30E-03	1,82E-02	
cg06620896	0,20	0,32	1,44E-02	2,51E-02	
cg17371203	0,69	0,59	2,18E-02	3,55E-02	SEMA6B
cg19070079	0,67	0,56	1,16E-02	1,79E-02	LOC101926975
cg04315336	0,64	0,52	2,92E-02	3,11E-02	THSD4
cg10777462	0,57	0,43	3,11E-03	1,37E-02	

cg27211179	0,58	0,47	3,86E-02	1,90E-02	EXD2
cg13314012	0,72	0,60	3,86E-02	5,78E-02	LINC01170
cg10953326	0,66	0,54	7,39E-03	1,59E-02	
cg19910976	0,63	0,52	1,60E-02	3,01E-02	
cg24868292	0,63	0,50	1,30E-02	2,85E-02	DNASE2B
cg24765446	0,55	0,44	3,21E-02	4,15E-02	WFDC6
cg13244293	0,40	0,30	1,02E-03	7,91E-03	NGLY1; OXSM
cg19664838	0,81	0,67	3,52E-02	3,49E-02	
cg15522953	0,74	0,58	5,52E-04	3,25E-03	ZMIZ1
cg10807084	0,44	0,56	4,61E-02	3,97E-02	AK5
cg20045300	0,60	0,46	1,97E-02	2,30E-02	FAM114A1
cg05545915	0,56	0,72	1,30E-02	3,09E-02	
cg10350446	0,65	0,54	9,30E-03	1,79E-02	
cg07035145	0,07	0,24	1,82E-03	3,18E-03	PYGL
cg16247085	0,47	0,37	3,52E-02	7,78E-02	LSAMP
cg02855302	0,63	0,52	4,22E-02	4,79E-02	CDC42BPB
cg14022913	0,44	0,31	2,65E-02	6,26E-02	NEIL3
cg09039163	0,59	0,70	4,22E-02	6,63E-02	ESPNL
cg16333846	0,70	0,59	2,18E-02	2,43E-02	TIAM2
cg16172329	0,76	0,62	6,57E-03	1,64E-02	DPP4
cg23633671	0,64	0,52	2,39E-03	1,25E-02	
cg21108805	0,39	0,27	1,58E-03	9,57E-03	WIF1
cg01334432	0,63	0,52	4,61E-02	9,04E-02	LOC642587; MIR205
cg14678654	0,47	0,58	1,60E-02	3,44E-02	
cg03522150	0,64	0,53	1,60E-02	4,42E-02	C11orf49
cg02772619	0,72	0,61	9,30E-03	2,54E-02	
cg18818644	0,68	0,56	1,97E-02	3,35E-02	
cg02078468	0,81	0,69	9,30E-03	1,26E-02	CERS2
cg12728465	0,59	0,47	1,30E-02	1,98E-02	ZKSCAN1
cg05867326	0,52	0,41	3,86E-02	3,75E-02	
cg23925996	0,20	0,30	2,18E-02	1,96E-02	ARL5B
cg19839026	0,26	0,41	2,18E-02	2,20E-02	PYGL
cg13905475	0,73	0,61	3,52E-02	2,41E-02	
cg22663660	0,69	0,55	6,57E-03	1,42E-02	LIPC
cg09829164	0,50	0,38	3,52E-02	3,29E-02	RADIL
cg10443977	0,86	0,74	1,30E-02	2,58E-02	ATP13A4
cg12178501	0,70	0,55	3,54E-03	9,82E-03	PLEKHS1
cg08514385	0,55	0,66	7,39E-03	4,61E-02	
cg03185290	0,68	0,55	5,16E-03	1,54E-02	CNOT4
cg07220152	0,11	0,22	5,16E-03	6,08E-02	
cg10640852	0,32	0,20	2,73E-03	1,44E-02	
cg10310395	0,86	0,69	2,18E-02	1,44E-02	TCF12
cg01766699	0,80	0,59	2,73E-03	7,58E-03	DNAH7
cg13469034	0,78	0,68	2,92E-02	2,72E-02	KIAA1026
cg16928392	0,88	0,74	5,16E-03	2,62E-02	DGKD

cg13742924	0,68	0,57	1,04E-02	2,34E-02	MARCH1
cg27488252	0,64	0,49	7,55E-04	1,18E-02	CLASP1
cg02820058	0,64	0,52	1,44E-02	3,18E-02	PRKG1
cg09684557	0,77	0,64	1,16E-02	1,50E-02	OR6C68
cg15851515	0,69	0,58	1,16E-02	2,61E-02	AIM1
cg04955791	0,53	0,43	3,21E-02	7,26E-02	SYNE1
cg12945572	0,61	0,48	1,58E-03	9,25E-03	PTGER3
cg05073607	0,56	0,44	1,16E-02	1,55E-02	POU6F2-AS1
cg20494691	0,54	0,40	1,18E-03	5,33E-03	MYCT1
cg18720720	0,44	0,31	4,56E-03	2,06E-02	
cg10028440	0,82	0,68	3,11E-03	1,66E-02	
cg03969251	0,59	0,49	2,39E-03	7,54E-03	DCLK2
cg10159032	0,75	0,63	9,30E-03	1,92E-02	LRRC37B
cg07954193	0,79	0,64	3,52E-02	4,79E-02	C1D
cg17457832	0,86	0,75	3,52E-02	3,12E-02	RPA3; UMAD1
cg01322839	0,71	0,55	1,58E-03	5,15E-03	CORO2A
cg11192416	0,23	0,41	1,97E-02	2,64E-02	
cg19080846	0,85	0,75	1,18E-03	1,59E-02	NRAP
cg24156694	0,55	0,44	7,39E-03	1,91E-02	UMODL1
cg25598696	0,63	0,49	4,56E-03	9,33E-03	MARCH1
cg19459178	0,67	0,51	2,65E-02	2,40E-02	PCSK6
cg18184895	0,84	0,73	1,78E-02	1,72E-02	MALRD1
cg13525835	0,85	0,73	8,30E-03	1,50E-02	CABLES1
cg16284880	0,52	0,37	1,60E-02	2,96E-02	
cg21456581	0,67	0,54	4,61E-02	3,12E-02	
cg05952081	0,16	0,32	4,61E-02	1,62E-02	
cg10191005	0,56	0,44	4,22E-02	1,74E-02	DIO3OS
cg24168276	0,77	0,66	2,65E-02	4,84E-02	BCL2
cg01160882	0,45	0,59	1,18E-03	7,93E-03	DKK1
cg14836363	0,77	0,65	3,21E-02	1,90E-02	
cg24435747	0,56	0,43	2,92E-02	3,28E-02	IL28RA
cg20823324	0,75	0,63	1,04E-02	2,84E-02	TMED3
cg14707974	0,39	0,50	2,18E-02	2,98E-02	
cg07867420	0,62	0,48	1,78E-02	1,89E-02	COL23A1
cg00869372	0,67	0,57	2,92E-02	2,84E-02	
cg01942816	0,58	0,47	1,78E-02	3,36E-02	MIR589
cg07498415	0,35	0,46	1,78E-02	1,90E-02	
cg01713201	0,62	0,47	5,83E-03	5,71E-03	SYNE1
cg04402745	0,74	0,62	6,57E-03	1,62E-02	
cg06072309	0,51	0,41	4,61E-02	4,46E-02	VIPR1
cg18162875	0,68	0,57	7,39E-03	1,01E-02	LOC100128531; KIAA1671
cg06113001	0,55	0,44	2,92E-02	2,28E-02	GLYCAM1
cg10664737	0,57	0,69	3,86E-02	4,60E-02	
cg23167136	0,73	0,59	3,21E-02	2,46E-02	CSRP1

cg21203643	0,67	0,56	1,30E-02	1,31E-02	DEGS2
cg18401380	0,63	0,52	2,18E-02	4,18E-02	MYO5B
cg17934874	0,78	0,64	6,57E-03	1,28E-02	AGAP1
cg19644270	0,65	0,55	2,65E-02	2,15E-02	
cg04931238	0,47	0,33	2,73E-03	1,20E-02	
cg05607305	0,75	0,62	1,37E-03	6,93E-03	
cg04440724	0,83	0,70	2,92E-02	2,34E-02	UBN1
cg15944290	0,51	0,39	2,92E-02	6,37E-02	MACROD2
cg26503877	0,17	0,31	1,16E-02	1,47E-02	ETS1
cg03755420	0,51	0,40	2,18E-02	2,30E-02	
cg14599421	0,69	0,54	1,82E-03	8,56E-03	DTNB
cg11832805	0,54	0,69	7,39E-03	1,71E-02	LOC284933; FAM19A5
cg15736126	0,74	0,56	8,79E-04	5,55E-03	ZNF296
cg22625504	0,49	0,38	2,18E-02	4,49E-02	TTI1
cg05026097	0,83	0,73	6,57E-03	1,70E-02	OTUD3
cg20954746	0,82	0,66	7,55E-04	5,28E-03	DGKD
cg08410479	0,70	0,59	9,30E-03	1,41E-02	LOC100128531; KIAA1671
cg25472135	0,81	0,71	1,78E-02	1,67E-02	SYNE3
cg01868551	0,49	0,36	1,97E-02	1,93E-02	
cg05895353	0,74	0,84	2,41E-02	7,96E-02	
cg07035704	0,62	0,51	2,18E-02	5,07E-02	MIR2052
cg24446969	0,54	0,40	4,56E-03	1,31E-02	NRXN3
cg20943095	0,62	0,50	1,97E-02	2,43E-02	CPLX3
cg08712054	0,34	0,23	2,92E-02	3,16E-02	HOXC4
cg25460263	0,70	0,58	3,11E-03	7,68E-03	XIRP2
cg23905763	0,23	0,38	3,86E-02	2,64E-02	SOGA3
cg12393334	0,69	0,55	2,65E-02	2,26E-02	
cg09765987	0,70	0,54	5,83E-03	1,54E-02	
cg06204665	0,65	0,54	4,22E-02	4,69E-02	NIPAL3
cg00670438	0,27	0,44	2,65E-02	1,87E-02	PYGL
cg08494221	0,42	0,52	3,86E-02	2,68E-02	PURG; WRN
cg08144021	0,74	0,64	1,60E-02	3,54E-02	CRTAC1
cg14483244	0,16	0,26	3,52E-02	3,94E-02	HDAC5
cg13466956	0,46	0,60	1,78E-02	2,70E-02	SLC19A3
cg03458344	0,55	0,43	2,92E-02	4,94E-02	C1orf129
cg14251889	0,57	0,47	1,97E-02	4,82E-02	KIF26B
cg05499811	0,61	0,49	1,30E-02	1,56E-02	DOCK8
cg17130176	0,78	0,67	1,16E-02	4,35E-02	BEST3
cg18902109	0,66	0,56	4,22E-02	6,57E-02	
cg26400135	0,80	0,69	3,52E-02	8,44E-02	
cg15808668	0,42	0,55	6,57E-03	1,77E-02	IP6K3
cg05585556	0,80	0,66	1,30E-02	1,65E-02	SNHG4
cg18593045	0,60	0,49	2,18E-02	3,00E-02	
cg23161350	0,59	0,49	1,78E-02	2,67E-02	

cg10141151	0,43	0,31	2,65E-02	4,82E-02	
cg09129997	0,47	0,35	1,37E-03	1,02E-02	
cg22076912	0,61	0,49	1,30E-02	1,63E-02	
cg15285104	0,78	0,63	9,30E-03	1,58E-02	LINC00160
cg19505827	0,31	0,45	2,18E-02	3,51E-02	TSPAN32
cg23313755	0,64	0,53	9,30E-03	1,53E-02	
cg20276367	0,69	0,56	1,58E-03	1,21E-02	
cg18086978	0,47	0,57	4,22E-02	3,92E-02	CLMN
cg09872392	0,60	0,48	1,78E-02	1,74E-02	PHKG1
cg14273219	0,68	0,55	4,56E-03	1,46E-02	PPP1R3F
cg17436674	0,52	0,38	1,19E-02	3,13E-02	ARHGEF38
cg12802876	0,50	0,39	4,61E-02	6,10E-02	
cg10528395	0,61	0,48	2,41E-02	1,69E-02	
cg05006873	0,50	0,39	2,18E-02	4,19E-02	
cg19914513	0,82	0,69	5,83E-03	7,38E-03	TTC12
cg17237907	0,61	0,71	3,86E-02	9,04E-02	MICAL1
cg17975941	0,57	0,47	4,22E-02	4,62E-02	
cg05865493	0,67	0,54	7,39E-03	1,47E-02	HDAC9
cg13686214	0,63	0,50	7,55E-04	5,49E-03	
cg08817204	0,66	0,54	2,18E-02	4,97E-02	NRG3
cg11345968	0,83	0,72	3,86E-02	6,59E-02	PRNCR1
cg03079840	0,63	0,47	2,02E-04	5,73E-03	SLC35F3
cg02136949	0,72	0,57	5,83E-03	2,41E-02	TRAPPC9
cg13762927	0,72	0,61	8,30E-03	1,36E-02	KIAA1456
cg12026858	0,69	0,57	3,86E-02	4,45E-02	ZBTB16
cg14503204	0,75	0,64	2,65E-02	3,68E-02	SGCZ
cg11924659	0,56	0,67	7,39E-03	4,90E-02	
cg06787433	0,79	0,66	2,18E-02	2,96E-02	SH3BP4
cg14707834	0,47	0,62	1,60E-02	1,63E-02	SIGIRR
cg13563869	0,55	0,45	1,30E-02	3,02E-02	
cg23863184	0,84	0,72	2,41E-02	3,57E-02	ZNF407
cg02512586	0,53	0,41	1,97E-02	3,63E-02	
cg00339415	0,46	0,36	2,18E-02	2,43E-02	OBP2B
cg14850831	0,74	0,62	2,41E-02	2,94E-02	GUCD1
cg22387864	0,64	0,49	1,30E-02	1,70E-02	ISPD
cg09683533	0,76	0,63	2,65E-02	2,30E-02	AFF1
cg00851827	0,74	0,59	8,30E-03	2,17E-02	ATP6V0D1
cg01635228	0,70	0,58	2,41E-02	2,20E-02	
cg22859289	0,33	0,43	3,52E-02	5,03E-02	
cg06526020	0,87	0,76	4,22E-02	3,05E-02	NUDT3
cg10652722	0,30	0,44	2,41E-02	2,78E-02	
cg11988321	0,55	0,66	4,22E-02	7,92E-02	MZB1
cg07248586	0,50	0,38	2,41E-02	2,63E-02	
cg19309009	0,62	0,49	3,54E-03	1,24E-02	MARK4
cg11737879	0,78	0,67	5,83E-03	1,43E-02	PEX3

cg23263302	0,64	0,52	3,86E-02	3,43E-02	LDB2
cg01708617	0,62	0,72	5,16E-03	1,24E-02	RAI14
cg01039497	0,57	0,46	2,41E-02	3,36E-02	TRAK1
cg01526396	0,47	0,58	2,92E-02	3,30E-02	
cg13919631	0,69	0,56	2,41E-02	3,60E-02	
cg14185504	0,60	0,46	1,16E-02	1,70E-02	FIG4
cg19698472	0,70	0,57	2,41E-02	1,88E-02	
cg18170393	0,69	0,58	3,21E-02	3,32E-02	
cg06261500	0,74	0,60	5,16E-03	1,21E-02	SND1
cg18962562	0,56	0,44	1,44E-02	2,25E-02	PPFIBP2
cg05135791	0,56	0,44	1,97E-02	4,00E-02	TRHDE
cg26656452	0,81	0,71	9,30E-03	1,36E-02	HABP2
cg00864666	0,56	0,43	2,39E-03	6,39E-03	RAPGEF4
cg00364758	0,62	0,47	1,78E-02	1,03E-02	EPDR1
cg23204757	0,26	0,15	7,39E-03	2,22E-02	
cg03885271	0,14	0,31	4,02E-03	3,04E-02	BHLHE23
cg15387301	0,77	0,65	1,82E-03	1,52E-02	RASGEF1B
cg25641388	0,69	0,56	3,21E-02	2,45E-02	
cg23470770	0,64	0,52	3,21E-02	4,32E-02	MAP3K5
cg01913492	0,69	0,57	3,86E-02	2,47E-02	
cg06036471	0,33	0,46	9,30E-03	1,62E-02	CPEB3
cg12966404	0,62	0,52	2,65E-02	4,96E-02	STRIP1
cg24464067	0,83	0,72	1,04E-02	3,39E-02	NET1
cg08473326	0,45	0,57	9,30E-03	1,66E-02	
cg14221048	0,79	0,61	1,41E-04	7,02E-03	
cg10308445	0,79	0,63	6,57E-03	8,51E-03	PGAP1
cg25668563	0,63	0,47	9,30E-03	1,98E-02	CCDC129
cg10085153	0,81	0,67	2,39E-03	6,03E-03	PAPSS2
cg06571387	0,50	0,61	6,57E-03	2,07E-02	HOXD12
cg21635588	0,67	0,57	3,21E-02	3,59E-02	IPCEF1
cg09974610	0,76	0,56	1,18E-03	4,43E-03	EHF
cg20137977	0,65	0,54	4,56E-03	1,30E-02	MCC
cg27484582	0,66	0,55	3,52E-02	3,57E-02	
cg18702947	0,81	0,66	8,30E-03	1,16E-02	PPFIA2
cg04761450	0,63	0,52	2,41E-02	2,40E-02	FOXO1
cg11028738	0,57	0,40	8,30E-03	1,58E-02	
cg15783703	0,86	0,74	1,78E-02	1,47E-02	AUTS2
cg02017534	0,31	0,43	4,22E-02	3,13E-02	SIAH3
cg19801921	0,42	0,57	2,92E-02	3,81E-02	
cg02361126	0,62	0,50	3,21E-02	3,57E-02	
cg06588136	0,82	0,71	4,71E-04	1,79E-02	CFH
cg01827197	0,64	0,53	1,97E-02	5,47E-02	A1CF
cg18202122	0,54	0,39	1,82E-03	5,74E-03	
cg09324866	0,55	0,44	1,41E-04	4,89E-03	
cg10308680	0,54	0,41	1,60E-02	2,24E-02	CCDC33

cg26581860	0,64	0,48	3,86E-02	3,78E-02	EHF
cg22596683	0,69	0,51	4,56E-03	1,04E-02	EHF
cg25865597	0,48	0,36	1,78E-02	2,44E-02	PRDM16
cg15167311	0,58	0,46	1,82E-03	9,13E-03	PRKD1
cg06231121	0,45	0,35	2,73E-03	1,70E-02	
cg13138848	0,66	0,51	3,54E-03	5,63E-03	
cg02723558	0,75	0,57	1,04E-02	1,02E-02	BDNFOS
cg10142997	0,52	0,41	1,60E-02	3,62E-02	PCDH9
cg20433989	0,59	0,49	1,97E-02	2,16E-02	FNDC3A
cg05719445	0,52	0,41	7,55E-04	4,98E-03	
cg06559333	0,76	0,56	2,08E-03	5,74E-03	AUTS2
cg04335144	0,63	0,52	1,04E-02	1,68E-02	PLEKHA7
cg12741293	0,65	0,44	2,39E-03	4,99E-03	
cg12678421	0,73	0,59	4,71E-04	8,98E-03	
cg16129213	0,82	0,69	8,30E-03	1,56E-02	SOD1
cg21187597	0,67	0,55	1,78E-02	2,86E-02	
cg25029197	0,62	0,47	2,39E-03	1,30E-02	KRT7
cg01204296	0,51	0,39	1,16E-02	2,94E-02	
cg04788216	0,74	0,56	1,44E-02	1,28E-02	EHF
cg17937897	0,53	0,36	4,41E-05	4,39E-03	
cg27130993	0,51	0,38	1,16E-02	1,41E-02	ABLIM3
cg04061534	0,58	0,47	7,39E-03	3,32E-02	RIN2
cg11581475	0,64	0,53	4,61E-02	3,90E-02	ZNF428
cg22509679	0,70	0,59	7,39E-03	1,65E-02	
cg25801428	0,60	0,49	1,97E-02	2,21E-02	LOC100132735
cg19015537	0,58	0,44	6,47E-04	6,18E-03	
cg01687997	0,68	0,53	3,54E-03	1,41E-02	C7orf50
cg20562823	0,63	0,51	1,30E-02	2,47E-02	
cg19612574	0,72	0,61	8,30E-03	2,60E-02	MAPK8
cg24716193	0,77	0,59	5,52E-04	9,37E-03	CASC21
cg27391744	0,72	0,56	7,55E-04	3,67E-03	AQP5; LOC101927318
cg21356379	0,81	0,69	1,16E-02	1,66E-02	
cg06571982	0,72	0,59	9,30E-03	1,33E-02	MRPL1
cg17585138	0,64	0,53	4,02E-03	1,28E-02	
cg03604322	0,52	0,40	2,92E-02	8,29E-02	SLC5A1
cg23900752	0,79	0,67	1,04E-02	1,49E-02	
cg25647648	0,70	0,51	1,37E-03	6,60E-03	MTHFD2P1
cg15069417	0,69	0,50	2,73E-03	5,40E-03	LOC100128531; KIAA1671
cg11020360	0,66	0,55	2,08E-03	2,91E-02	LCP2; C5orf58
cg10316857	0,76	0,63	9,30E-03	1,23E-02	
cg09366829	0,61	0,47	1,04E-02	1,10E-02	VAV3
cg25754826	0,46	0,35	2,73E-03	9,24E-03	AUTS2
cg01212975	0,78	0,67	2,41E-02	2,02E-02	
cg08771884	0,66	0,54	9,30E-03	1,89E-02	UBN1

cg10549234	0,74	0,59	4,02E-03	8,76E-03	ADGRL2
cg01892746	0,49	0,36	2,65E-02	3,79E-02	SYNE1
cg03692576	0,52	0,41	1,82E-03	9,78E-03	
cg18041719	0,40	0,27	3,39E-04	6,15E-03	SEMA3D
cg21687351	0,61	0,50	1,78E-02	1,96E-02	KERA
cg26479374	0,49	0,37	3,21E-02	5,01E-02	CTBP1
cg13721302	0,77	0,59	1,58E-03	9,27E-03	SRPK2
cg17608436	0,60	0,48	7,39E-03	1,49E-02	
cg14930320	0,53	0,40	2,41E-02	2,86E-02	
cg18284319	0,71	0,56	3,52E-02	2,28E-02	RNF4
cg13523510	0,47	0,37	3,86E-02	3,66E-02	
cg15471172	0,70	0,55	1,44E-02	1,27E-02	
cg08614769	0,77	0,63	3,21E-02	2,16E-02	GMDS
cg09337641	0,70	0,59	3,52E-02	4,46E-02	
cg20636399	0,58	0,47	7,39E-03	1,86E-02	SLC24A4
cg13984883	0,65	0,54	3,11E-03	1,62E-02	AP1B1
cg18181545	0,50	0,39	1,30E-02	1,75E-02	EDIL3
cg00461650	0,45	0,34	2,41E-02	3,30E-02	
cg25134328	0,34	0,45	3,21E-02	3,83E-02	
cg05966408	0,56	0,45	5,83E-03	1,62E-02	NRG2
cg14613491	0,56	0,46	1,44E-02	2,80E-02	CNTN5
cg17043875	0,68	0,53	1,04E-02	1,33E-02	
cg07737938	0,60	0,47	5,83E-03	1,37E-02	
cg20350898	0,56	0,43	6,57E-03	1,13E-02	SIKE1
cg15270502	0,67	0,55	4,02E-03	1,42E-02	
cg00550493	0,68	0,56	2,65E-02	1,98E-02	EYS
cg13832372	0,39	0,53	1,04E-02	1,56E-02	LHX6
cg12121765	0,77	0,66	3,21E-02	3,24E-02	DLC1
cg09334240	0,58	0,46	2,65E-02	3,28E-02	ATF7
cg02909298	0,69	0,53	5,16E-03	8,07E-03	
cg08765573	0,49	0,37	1,30E-02	1,99E-02	SLC35F3
cg12452334	0,73	0,60	1,60E-02	2,10E-02	
cg05179455	0,49	0,38	4,22E-02	6,77E-02	COL6A3
cg05438832	0,63	0,53	1,30E-02	2,62E-02	
cg11266682	0,31	0,21	1,78E-02	2,54E-02	SLC2A9
cg11666559	0,27	0,40	1,44E-02	2,11E-02	
cg05801088	0,35	0,50	3,86E-02	2,17E-02	
cg26440512	0,63	0,52	7,39E-03	2,02E-02	ABLIM2
cg01541932	0,71	0,57	2,92E-02	3,36E-02	CTNNA2
cg19931596	0,66	0,51	3,54E-03	1,13E-02	SLC25A12
cg26640276	0,60	0,46	1,30E-02	1,83E-02	
cg03928546	0,19	0,35	1,97E-02	2,19E-02	CCDC105
cg12602197	0,47	0,36	3,11E-03	3,62E-02	
cg05293827	0,57	0,43	8,30E-03	2,86E-02	PDE1C
cg14219442	0,68	0,55	1,04E-02	1,11E-02	

cg08995655	0,67	0,55	1,30E-02	2,64E-02	NUDT19
cg18290075	0,15	0,27	4,61E-02	7,42E-02	SGEF
cg07229402	0,58	0,44	5,83E-03	1,60E-02	
cg21948636	0,74	0,59	2,73E-03	1,13E-02	
cg01986648	0,56	0,42	1,16E-02	2,20E-02	
cg13075537	0,58	0,47	1,60E-02	2,65E-02	TMEM57
cg25266629	0,30	0,47	2,65E-02	3,32E-02	TLX1
cg07145899	0,71	0,61	4,02E-03	1,25E-02	OTUD3
cg15496665	0,69	0,57	1,97E-02	3,88E-02	PAPPA
cg10070202	0,64	0,53	9,30E-03	2,82E-02	OSBP2
cg04230537	0,82	0,71	6,57E-03	1,27E-02	TRAP1
cg19828122	0,64	0,51	1,16E-02	1,13E-02	VAV3
cg13169408	0,50	0,39	9,30E-03	2,00E-02	
cg09161311	0,69	0,55	9,30E-03	1,24E-02	
cg12204957	0,77	0,62	4,00E-04	9,32E-03	IRAK2
cg11305739	0,65	0,50	1,44E-02	1,64E-02	
cg02510157	0,65	0,51	7,39E-03	1,23E-02	PDE4B
cg08549781	0,61	0,48	2,41E-02	2,59E-02	AP1S3
cg11406977	0,57	0,45	2,65E-02	3,15E-02	
cg05562252	0,80	0,70	4,61E-02	2,65E-02	CD164
cg00574062	0,74	0,62	2,92E-02	4,81E-02	ASIC2
cg19177771	0,48	0,37	4,61E-02	3,18E-02	
cg26127201	0,70	0,57	3,21E-02	2,96E-02	
cg07461712	0,72	0,52	2,39E-03	6,33E-03	LRRC37B
cg09838568	0,20	0,34	3,21E-02	2,01E-02	
cg17053016	0,66	0,56	3,21E-02	5,13E-02	
cg17230414	0,66	0,53	2,65E-02	1,98E-02	
cg15937958	0,73	0,53	1,02E-03	8,32E-03	CXCL17
cg07721027	0,71	0,59	2,39E-03	1,70E-02	IP6K3
cg19958586	0,49	0,59	3,21E-02	3,76E-02	CCRL2
cg20791945	0,77	0,66	2,08E-03	8,23E-03	
cg27452462	0,78	0,68	2,65E-02	2,81E-02	LINC01169
cg15707869	0,66	0,55	9,30E-03	1,83E-02	DCAF4L2
cg23558626	0,75	0,63	4,61E-02	2,35E-02	
cg17726317	0,56	0,41	3,52E-02	2,51E-02	
cg13138556	0,74	0,62	1,44E-02	2,21E-02	ENPP6
cg14936543	0,54	0,43	2,65E-02	2,45E-02	AGBL1
cg26357982	0,66	0,54	2,41E-02	2,27E-02	SLCO1B7
cg25182785	0,67	0,51	1,82E-03	8,64E-03	ARHGEF28
cg20522398	0,52	0,39	1,97E-02	4,18E-02	
cg23010084	0,63	0,49	1,37E-03	7,08E-03	ADGRB3
cg14015502	0,55	0,42	3,35E-02	2,77E-02	C10orf26
cg06026636	0,70	0,55	4,56E-03	9,05E-03	SND1
cg25322618	0,49	0,37	2,65E-02	2,93E-02	RAPGEF4
cg14474138	0,71	0,53	9,30E-03	1,19E-02	

cg13884444	0,55	0,43	1,18E-03	8,74E-03	SUN3
cg03277898	0,77	0,65	2,92E-02	2,55E-02	
cg15800767	0,33	0,46	7,39E-03	2,35E-02	LOC100128770; CCDC64B
cg14927641	0,55	0,45	1,30E-02	5,40E-02	
cg16541681	0,65	0,50	5,40E-05	2,74E-03	IPCEF1
cg03140412	0,45	0,63	7,39E-03	2,26E-02	SIGIRR
cg16744531	0,39	0,21	3,54E-03	1,86E-02	B3GNT3
cg24011522	0,77	0,67	5,83E-03	2,73E-02	
cg03691573	0,24	0,39	4,61E-02	1,55E-02	LOC100130451; SPAG16
cg11898646	0,40	0,52	9,30E-03	3,51E-02	EBF1
cg20543968	0,64	0,48	2,39E-03	8,57E-03	
cg24335252	0,40	0,56	2,65E-02	2,34E-02	
cg14337655	0,24	0,38	1,18E-03	8,72E-03	C19orf35
cg09641663	0,82	0,72	1,18E-03	9,89E-03	OSBPL10
cg06058203	0,68	0,54	2,08E-03	8,28E-03	MECP2
cg16770641	0,61	0,49	2,18E-02	2,34E-02	
cg19261904	0,77	0,63	2,18E-02	1,72E-02	
cg15008149	0,57	0,42	1,18E-03	6,04E-03	LOC100128531; KIAA1671
cg03251454	0,51	0,41	4,22E-02	6,16E-02	
cg26198638	0,73	0,57	3,54E-03	1,12E-02	
cg19795338	0,58	0,43	1,18E-03	9,12E-03	ATP13A4
cg20343746	0,69	0,55	2,18E-02	2,71E-02	GGACT
cg08486363	0,59	0,47	1,30E-02	2,69E-02	LINC01587
cg04919036	0,53	0,43	5,16E-03	4,89E-02	
cg02271687	0,65	0,53	1,60E-02	2,45E-02	PIK3R1
cg25094495	0,50	0,39	1,60E-02	4,26E-02	
cg06638913	0,72	0,60	1,30E-02	1,73E-02	SH3BP4
cg07842960	0,57	0,42	1,04E-02	1,52E-02	SH3BP4
cg06477323	0,69	0,54	4,71E-04	6,63E-03	PRKCH
cg13660662	0,51	0,40	2,39E-03	1,86E-02	
cg07902983	0,30	0,42	2,41E-02	4,17E-02	SYCN
cg12766569	0,74	0,63	2,92E-02	2,67E-02	KLK11
cg02065027	0,80	0,69	4,61E-02	2,41E-02	ARHGEF12
cg26680428	0,50	0,37	4,61E-02	4,66E-02	
cg09802076	0,42	0,29	1,97E-02	3,20E-02	SLC6A15
cg25838080	0,51	0,61	2,65E-02	2,53E-02	NR2E1
cg13623999	0,66	0,55	3,21E-02	2,03E-02	
cg01178013	0,80	0,69	1,78E-02	1,84E-02	TANC1
cg07009554	0,45	0,56	1,30E-02	3,74E-02	
cg05181918	0,56	0,43	3,54E-03	8,62E-03	LOC100128531; KIAA1671
cg22800453	0,61	0,49	1,97E-02	1,05E-02	MUCL1
cg02320543	0,54	0,66	1,97E-02	3,99E-02	

cg20816107	0,54	0,40	4,02E-03	1,32E-02	PLCL1
cg17672195	0,50	0,37	1,97E-02	2,41E-02	LOC100507205
cg24546683	0,43	0,31	4,02E-03	2,44E-02	
cg02315271	0,41	0,54	4,61E-02	5,24E-02	
cg01915989	0,60	0,39	1,02E-03	9,41E-03	
cg04188392	0,61	0,48	2,39E-03	1,19E-02	TUSC8
cg18816129	0,71	0,59	2,73E-03	1,09E-02	MEIS2
cg11723489	0,54	0,43	1,30E-02	3,44E-02	
cg08349093	0,64	0,53	1,97E-02	2,84E-02	
cg00889540	0,53	0,43	1,44E-02	3,02E-02	
cg23321751	0,56	0,42	5,16E-03	1,75E-02	KIAA1328
cg27451581	0,67	0,51	1,44E-02	1,53E-02	KLHDC7A
cg06227673	0,65	0,53	1,37E-03	7,08E-03	
cg15948428	0,46	0,57	4,61E-02	6,06E-02	
cg08638124	0,71	0,59	3,21E-02	3,82E-02	ASB2
cg09766556	0,61	0,48	1,60E-02	2,31E-02	SGCZ
cg06957219	0,64	0,51	1,97E-02	2,99E-02	DISC1
cg25433259	0,43	0,32	2,39E-03	9,36E-03	OSTCP1
cg11137178	0,90	0,80	7,39E-03	4,84E-02	
cg01689653	0,67	0,54	1,58E-03	1,07E-02	ABR
cg13092992	0,74	0,60	4,56E-03	1,13E-02	
cg18034674	0,37	0,53	3,86E-02	2,19E-02	
cg07825247	0,71	0,58	1,30E-02	1,79E-02	STAG1
cg24843615	0,56	0,42	1,16E-02	1,68E-02	MSGN1
cg16698721	0,53	0,42	3,86E-02	5,88E-02	CERS4
cg15202552	0,44	0,33	3,54E-03	2,04E-02	FCRL3
cg13888854	0,48	0,59	1,97E-02	2,75E-02	IL1R2
cg13469457	0,61	0,49	2,92E-02	3,80E-02	
cg03389928	0,64	0,52	3,21E-02	2,99E-02	KCNQ5
cg16677112	0,62	0,46	2,08E-03	7,28E-03	EFNA1
cg25793657	0,77	0,66	3,86E-02	2,46E-02	SNHG4; SNORA74A; MATR3
cg01518970	0,69	0,57	6,57E-03	1,12E-02	
cg04675946	0,64	0,53	3,21E-02	4,29E-02	
cg23046990	0,72	0,57	2,92E-02	2,57E-02	LINC00578
cg03402807	0,76	0,62	2,41E-02	3,01E-02	PRICKLE2
cg25190663	0,63	0,51	7,39E-03	2,03E-02	
cg20988291	0,39	0,27	1,60E-02	3,09E-02	
cg09423378	0,77	0,60	1,58E-03	8,34E-03	ARHGEF28
cg10151281	0,64	0,47	8,30E-03	1,93E-02	TPM4
cg00223647	0,52	0,41	1,60E-02	2,26E-02	
cg05548017	0,62	0,52	3,86E-02	5,18E-02	FGD5
cg16688617	0,51	0,40	2,41E-02	3,68E-02	
cg01250864	0,59	0,44	1,37E-03	7,08E-03	IFNGR1
cg25378392	0,53	0,41	1,78E-02	2,84E-02	

cg11537026	0,74	0,57	2,41E-02	1,14E-02	
cg11351983	0,42	0,30	2,41E-02	1,99E-02	ESRRG
cg17401082	0,44	0,33	1,97E-02	3,51E-02	
cg17572723	0,67	0,57	1,97E-02	1,86E-02	ZNF767P
cg01214012	0,65	0,51	4,22E-02	3,53E-02	
cg12800585	0,58	0,48	8,30E-03	1,42E-02	ARHGAP35
cg15679007	0,62	0,50	2,92E-02	3,69E-02	
cg20832020	0,68	0,57	4,22E-02	4,78E-02	TIGIT
cg25765881	0,49	0,36	1,44E-02	2,31E-02	
cg18566313	0,50	0,33	4,02E-03	1,09E-02	SORBS2
cg09015280	0,10	0,21	3,21E-02	4,64E-02	
cg05881682	0,66	0,52	2,18E-02	3,43E-02	
cg19628619	0,66	0,51	4,02E-03	8,96E-03	EFNA1
cg25254263	0,81	0,65	1,82E-03	8,06E-03	C12orf79
cg26983411	0,64	0,52	3,21E-02	3,99E-02	
cg01613870	0,60	0,46	4,02E-03	1,06E-02	
cg15412918	0,64	0,53	2,65E-02	2,52E-02	
cg01439876	0,37	0,47	1,04E-02	1,93E-02	
cg12545864	0,48	0,35	1,44E-02	1,99E-02	
cg25640196	0,72	0,61	4,02E-03	8,64E-03	RAD51B

Supplementary table S2: DNA methylation age results for metEPICVal samples.

Sample	Chronological Age	DNAmAge	AgeAccelerationDiff	AgeAccelerationResidual
Met32	63	87,32	24,32	23,06
Met31	62	52,73	-9,27	-11,1
Met20	33	27,72	-5,28	-23,73
Met33	69	83,21	14,21	16,39
Met55	34	54,32	20,32	2,44
Met36	51	38,14	-12,86	-21
Met22	35	48,44	13,44	-3,87
Met21	34	53,86	19,86	1,98
Met38	59	60,99	1,99	-1,56
Met15	33	43,19	10,19	-8,27
Met37	66	85,17	19,17	19,64
Met23	28	43,47	15,47	-5,85
Met24	34	53,35	19,35	1,47
Met57	34	38,74	4,74	-13,14
Met39	67	72,46	5,46	6,5
Met42	72	54,15	-17,85	-13,95
Met26	34	40,58	6,58	-11,3
Met43	61	61,11	0,11	-2,28
Met28	31	61,39	30,39	10,79
Met46	81	54,41	-26,59	-17,52
Met34	69	67,99	-1,01	1,18
Met56	26	36,65	10,65	-11,82
Met29	33	36,68	3,68	-14,77
Met30	28	50,54	22,54	1,22
Met50	34	86,36	52,36	34,48
Met54	33	40,54	7,54	-10,92
Met53	34	59,98	25,98	8,1
Met51	34	44,98	10,98	-6,9
Met49	54	55,88	1,88	-4,54
Met61	28	64,57	36,57	15,25
Met60	35	70,69	35,69	18,39
Met63	33	75,23	42,23	23,77
Met40	77	71,2	-5,8	0,97
Met62	35	49,16	14,16	-3,15

Supplementary Table S3: Significant CpG probes localized in promotor regions from hypomethylation global profile in BCVY from metEPICVal study

Probes	UCSC RefGene Name	Methylation differences (BCVY - BCO)	Gene expression differences (BCVY - BCO)	*p value*	Adjusted *p-value*
cg20475650	EHD2	-0,14	0,47	1,10E-50	2,45E-49
cg01309726	LOC100270710	-0,13	0,46	1,40E-49	2,98E-48
cg10910579	TMEM59L	-0,16	0,79	2,25E-49	4,62E-48
cg11399100	TMEM59L	-0,15	0,79	2,25E-49	4,62E-48
cg17450859	ASB5	0,18	0,49	1,87E-48	3,74E-47
cg18342872	ASB5	0,18	0,49	1,87E-48	3,74E-47
cg08646125	LRRC3B	-0,12	1,04	9,24E-48	1,79E-46
cg26035366	LRRC3B	-0,17	1,04	9,24E-48	1,79E-46
cg06869039	C8orf79	0,13	1,05	2,70E-45	5,02E-44
cg01622379	LHX8	-0,17	-0,55	1,34E-43	2,25E-42
cg01185626	LHX8	-0,18	-0,55	1,34E-43	2,25E-42
cg25368122	LHX8	-0,24	-0,55	1,34E-43	2,25E-42
cg23952663	LHX8	-0,27	-0,55	1,34E-43	2,25E-42
cg19764599	LHX8	-0,21	-0,55	1,34E-43	2,25E-42
cg12764034	LHX8	-0,14	-0,55	1,34E-43	2,25E-42
cg20494691	MYCT1	0,14	0,58	6,87E-42	1,13E-40
cg23183497	GRM3	-0,15	0,46	3,20E-41	4,82E-40
cg17814180	GRM3	-0,18	0,46	3,20E-41	4,82E-40
cg00762869	GRM3	-0,25	0,46	3,20E-41	4,82E-40
cg15331781	GRM3	-0,14	0,46	3,20E-41	4,82E-40
cg11210312	GRM3	0,17	0,46	3,20E-41	4,82E-40
cg01005728	ABCB1	0,14	0,91	4,29E-40	5,69E-39
cg15759056	ABCB1	-0,29	0,91	4,29E-40	5,69E-39
cg25438493	ABCB1	-0,26	0,91	4,29E-40	5,69E-39
cg12270822	ABCB1	-0,20	0,91	4,29E-40	5,69E-39
cg00141548	ABCB1	-0,15	0,91	4,29E-40	5,69E-39
cg09105881	ABCB1	-0,17	0,91	4,29E-40	5,69E-39
cg27507700	ABCB1	-0,25	0,91	4,29E-40	5,69E-39
cg06562372	ABCB1	-0,19	0,91	4,29E-40	5,69E-39
cg00862116	ABCB1	-0,20	0,91	4,29E-40	5,69E-39
cg10126372	PXDNL	-0,10	-0,98	2,98E-38	3,69E-37
cg20383036	RHOJ	0,10	0,57	7,21E-38	8,78E-37
cg05350199	RHOJ	0,13	0,57	7,21E-38	8,78E-37
cg08479860	ADCY8	0,15	0,34	8,87E-38	1,06E-36
cg16696270	ADCY8	-0,13	0,34	8,87E-38	1,06E-36
cg19159488	CLEC14A	0,14	0,43	3,18E-34	3,22E-33
cg16482474	WDR86	-0,16	0,88	3,37E-34	3,37E-33
cg17509603	WDR86	-0,21	0,88	3,37E-34	3,37E-33

cg27304406	BNC1	-0,18	0,73	6,57E-30	6,02E-29
cg18560204	BNC1	-0,17	0,73	6,57E-30	6,02E-29
cg18952647	BNC1	-0,22	0,73	6,57E-30	6,02E-29
cg14385245	BNC1	-0,25	0,73	6,57E-30	6,02E-29
cg07224636	TMTC1	0,14	0,76	1,56E-28	1,38E-27
cg08729318	TMTC1	-0,12	0,76	1,56E-28	1,38E-27
cg01672943	PAX9	-0,27	-0,98	7,58E-25	5,97E-24
cg01972418	PAX9	-0,31	-0,98	7,58E-25	5,97E-24
cg01627823	PAX9	-0,35	-0,98	7,58E-25	5,97E-24
cg00970313	PAX9	-0,22	-0,98	7,58E-25	5,97E-24
cg23655970	PAX9	-0,19	-0,98	7,58E-25	5,97E-24
cg00388556	PAX9	-0,23	-0,98	7,58E-25	5,97E-24
cg21875330	PAX9	-0,24	-0,98	7,58E-25	5,97E-24
cg03938432	BAI3	-0,21	0,47	1,69E-24	1,30E-23
cg10244047	BAI3	-0,13	0,47	1,69E-24	1,30E-23
cg02653557	ZIK1	-0,17	0,62	4,05E-24	3,08E-23
cg01046104	ZIK1	-0,26	0,62	4,05E-24	3,08E-23
cg15343461	TMEM90B	-0,18	-0,98	1,62E-23	1,22E-22
cg12403137	NAALAD2	-0,23	0,48	2,11E-23	1,55E-22
cg05500015	NAALAD2	-0,24	0,48	2,11E-23	1,55E-22
cg09584827	NAALAD2	-0,25	0,48	2,11E-23	1,55E-22
cg14304817	NAALAD2	-0,19	0,48	2,11E-23	1,55E-22
cg10599900	REM1	-0,19	0,41	3,66E-23	2,67E-22
cg00741624	KIAA1409	-0,23	0,38	9,95E-23	7,19E-22
cg06622151	SIX3	-0,17	-0,94	2,93E-22	2,10E-21
cg21604358	HPCA	-0,19	0,44	4,43E-22	3,14E-21
cg11712990	LOC645323	-0,20	-0,77	3,14E-21	2,08E-20
cg19411025	LOC645323	-0,19	-0,77	3,14E-21	2,08E-20
cg04261138	LOC645323	-0,21	-0,77	3,14E-21	2,08E-20
cg12325536	LOC645323	-0,23	-0,77	3,14E-21	2,08E-20
cg08811309	LOC645323	-0,13	-0,77	3,14E-21	2,08E-20
cg16638920	LOC645323	-0,19	-0,77	3,14E-21	2,08E-20
cg24058566	LOC645323	-0,17	-0,77	3,14E-21	2,08E-20
cg09884341	LOC645323	-0,19	-0,77	3,14E-21	2,08E-20
cg01080998	LOC645323	-0,21	-0,77	3,14E-21	2,08E-20
cg20459527	OR13C2	0,18	0,06	8,13E-21	5,34E-20
cg23095584	GBX2	-0,21	-0,46	2,72E-20	1,77E-19
cg06953874	HOXD9	-0,14	0,43	3,31E-19	2,00E-18
cg10957151	HOXD9	-0,21	0,43	3,31E-19	2,00E-18
cg14142007	HOXD9	-0,23	0,43	3,31E-19	2,00E-18
cg22805974	HOXD9	-0,17	0,43	3,31E-19	2,00E-18
cg14991487	HOXD9	-0,21	0,43	3,31E-19	2,00E-18
cg09578028	HOXD9	-0,24	0,43	3,31E-19	2,00E-18
cg05167251	HOXD9	-0,24	0,43	3,31E-19	2,00E-18
cg19464917	ISL2	-0,11	-0,63	3,64E-19	2,18E-18

cg18961944	GALR1	-0,15	0,22	8,05E-19	4,55E-18
cg06562865	GALR1	-0,23	0,22	8,05E-19	4,55E-18
cg15484988	GALR1	-0,19	0,22	8,05E-19	4,55E-18
cg18664869	PCDHB15	-0,23	0,36	1,54E-17	8,04E-17
cg10757144	PCDHB15	-0,16	0,36	1,54E-17	8,04E-17
cg13298963	ACCN1	-0,10	0,97	1,90E-17	9,67E-17
cg25033990	ACCN1	-0,22	0,97	1,90E-17	9,67E-17
cg19942495	ACCN1	-0,19	0,97	1,90E-17	9,67E-17
cg24613080	ACCN1	-0,22	0,97	1,90E-17	9,67E-17
cg14603098	ACCN1	-0,20	0,97	1,90E-17	9,67E-17
cg17491850	DAB1	-0,16	0,30	1,97E-17	9,78E-17
cg24351410	DAB1	-0,17	0,30	1,97E-17	9,78E-17
cg21177673	DAB1	-0,16	0,30	1,97E-17	9,78E-17
cg26345888	DAB1	-0,16	0,30	1,97E-17	9,78E-17
cg11256607	DAB1	-0,20	0,30	1,97E-17	9,78E-17
cg06384463	BARHL2	-0,18	-0,19	5,39E-17	2,59E-16
cg06959142	BARHL2	-0,19	-0,19	5,39E-17	2,59E-16
cg13146465	BARHL2	-0,19	-0,19	5,39E-17	2,59E-16
cg17241310	BARHL2	-0,20	-0,19	5,39E-17	2,59E-16
cg12520861	OTP	-0,15	-0,44	1,15E-16	5,42E-16
cg21960680	OTP	-0,21	-0,44	1,15E-16	5,42E-16
cg08572394	OTP	-0,24	-0,44	1,15E-16	5,42E-16
cg12054820	OTP	-0,14	-0,44	1,15E-16	5,42E-16
cg05467523	SV2A	-0,13	0,54	3,34E-16	1,56E-15
cg15119027	FGF3	-0,26	0,03	6,21E-15	2,81E-14
cg24359323	FGF3	-0,21	0,03	6,21E-15	2,81E-14
cg15337897	FGF3	-0,24	0,03	6,21E-15	2,81E-14
cg20308540	PLD5	-0,11	0,63	1,06E-14	4,74E-14
cg22679728	PLD5	-0,18	0,63	1,06E-14	4,74E-14
cg12613383	PLD5	-0,18	0,63	1,06E-14	4,74E-14
cg03637878	JAM3	-0,20	0,36	4,31E-14	1,84E-13
cg00939226	JAM3	-0,23	0,36	4,31E-14	1,84E-13
cg24899571	JAM3	-0,16	0,36	4,31E-14	1,84E-13
cg02174225	JAM3	-0,17	0,36	4,31E-14	1,84E-13
cg14337655	C19orf35	-0,14	0,34	1,47E-13	6,22E-13
cg01970716	NYNRIN	-0,11	0,39	5,98E-13	2,50E-12
cg25824543	ZNF175	-0,19	0,35	6,54E-13	2,72E-12
cg15881486	CBR3	0,12	-0,38	6,81E-13	2,82E-12
cg16361890	LTC4S	-0,17	0,47	1,03E-12	4,24E-12
cg21176048	PEX5L	-0,25	0,39	2,42E-12	9,95E-12
cg20914464	NOL4	-0,15	0,69	5,92E-12	2,41E-11
cg22593533	OLIG2	-0,16	-0,19	1,07E-11	4,30E-11
cg20455092	GHR	-0,13	0,51	2,89E-11	1,14E-10
cg18409528	TLX2	-0,23	-0,22	3,99E-11	1,53E-10
cg03998104	TLX2	-0,25	-0,22	3,99E-11	1,53E-10

cg15112032	PCDH17	-0,22	-0,28	4,47E-11	1,70E-10
cg14427009	PCDH17	-0,17	-0,28	4,47E-11	1,70E-10
cg12398338	PVRL3	-0,19	0,47	5,51E-11	2,08E-10
cg00705142	PVRL3	-0,15	0,47	5,51E-11	2,08E-10
cg03654735	RSPO1	-0,21	0,66	7,11E-11	2,66E-10
cg01398050	KCNH8	-0,20	0,66	3,22E-10	1,18E-09
cg24997944	KCNH8	-0,21	0,66	3,22E-10	1,18E-09
cg24668089	KCNH8	-0,13	0,66	3,22E-10	1,18E-09
cg18500192	KCNH8	-0,27	0,66	3,22E-10	1,18E-09
cg04891086	KCNA1	-0,22	0,56	5,19E-10	1,87E-09
cg09487611	KCNA1	-0,13	0,56	5,19E-10	1,87E-09
cg15682043	KCNA1	-0,18	0,56	5,19E-10	1,87E-09
cg06204922	KCNA1	-0,26	0,56	5,19E-10	1,87E-09
cg16907029	KCNA1	-0,11	0,56	5,19E-10	1,87E-09
cg26162295	GSDMA	-0,11	-0,59	5,44E-10	1,95E-09
cg20699141	DCUN1D1	-0,14	-0,22	5,59E-10	2,00E-09
cg03895540	OR52N2	0,16	0,22	9,70E-10	3,45E-09
cg04360049	CD1D	-0,19	0,32	2,69E-08	9,08E-08
cg25683325	CD1D	-0,23	0,32	2,69E-08	9,08E-08
cg24900205	KCNT2	0,15	0,45	1,41E-07	4,57E-07
cg14664759	KCNT2	-0,16	0,45	1,41E-07	4,57E-07
cg09177518	KCNT2	-0,19	0,45	1,41E-07	4,57E-07
cg02858606	CDH8	-0,23	0,60	2,32E-07	7,14E-07
cg03671700	CDH8	-0,15	0,60	2,32E-07	7,14E-07
cg11323198	CDH8	-0,19	0,60	2,32E-07	7,14E-07
cg27120816	CDH8	-0,21	0,60	2,32E-07	7,14E-07
cg01718742	CDH8	-0,25	0,60	2,32E-07	7,14E-07
cg01007828	CDH8	-0,22	0,60	2,32E-07	7,14E-07
cg06830659	CDH8	-0,22	0,60	2,32E-07	7,14E-07
cg22465581	CDH8	-0,22	0,60	2,32E-07	7,14E-07
cg17995563	CDH8	-0,17	0,60	2,32E-07	7,14E-07
cg06321345	FAM5B	-0,13	0,89	3,62E-07	1,11E-06
cg19519310	FOXF2	-0,12	0,24	1,20E-06	3,63E-06
cg09246637	SNTG1	-0,19	0,01	1,56E-06	4,71E-06
cg00230631	SNTG1	-0,23	0,01	1,56E-06	4,71E-06
cg09238180	HOXD8	-0,21	0,37	2,48E-06	7,39E-06
cg20732478	HOXD8	-0,11	0,37	2,48E-06	7,39E-06
cg21815667	HOXD8	-0,21	0,37	2,48E-06	7,39E-06
cg27351358	BDNF	-0,17	0,54	3,44E-06	1,01E-05
cg16257091	BDNF	-0,20	0,54	3,44E-06	1,01E-05
cg03167496	BDNF	-0,19	0,54	3,44E-06	1,01E-05
cg22288103	BDNF	-0,22	0,54	3,44E-06	1,01E-05
cg25457956	BDNF	-0,26	0,54	3,44E-06	1,01E-05
cg15462887	BDNF	-0,23	0,54	3,44E-06	1,01E-05
cg25156688	BDNF	-0,25	0,54	3,44E-06	1,01E-05

cg01583131	BDNF	-0,15	0,54	3,44E-06	1,01E-05
cg17206583	VGF	-0,12	-0,38	9,19E-06	2,63E-05
cg12135573	VGF	-0,17	-0,38	9,19E-06	2,63E-05
cg12865837	SIM1	-0,20	-0,66	1,28E-05	3,62E-05
cg21063722	SIM1	-0,25	-0,66	1,28E-05	3,62E-05
cg11891393	SIM1	-0,17	-0,66	1,28E-05	3,62E-05
cg22826141	SFRP4	-0,15	-0,58	2,25E-05	6,28E-05
cg07669460	SFRP4	-0,18	-0,58	2,25E-05	6,28E-05
cg26678765	C1orf161	0,23	-0,40	2,77E-05	7,73E-05
cg07157893	MARCH1	0,16	-0,35	3,05E-05	8,46E-05
cg00032805	MARCH1	-0,14	-0,35	3,05E-05	8,46E-05
cg03557733	MPP2	-0,17	0,42	9,49E-05	2,60E-04
cg06304401	NLGN1	-0,23	0,29	9,68E-05	2,64E-04
cg24600895	NLGN1	-0,11	0,29	9,68E-05	2,64E-04
cg05754905	F11R	0,18	-0,17	1,15E-04	3,12E-04
cg04597433	DRD5	-0,15	0,02	1,18E-04	3,21E-04
cg14309111	ODZ4	-0,13	-0,36	1,37E-04	3,71E-04
cg10729426	ZNF549	-0,18	0,26	2,31E-04	6,12E-04
cg27304204	TSPAN8	0,19	0,80	3,39E-04	8,87E-04
cg15684563	TSPAN8	0,15	0,80	3,39E-04	8,87E-04
cg04950931	TSPAN8	0,12	0,80	3,39E-04	8,87E-04
cg19871235	TSPAN8	0,15	0,80	3,39E-04	8,87E-04
cg22387174	CNPY1	-0,20	0,05	3,63E-04	9,48E-04
cg09265529	ZNF470	-0,21	-0,19	5,06E-04	1,32E-03
cg27109600	FLJ32063	-0,16	0,09	7,48E-04	1,93E-03
cg22862420	RGS17	0,15	0,16	7,55E-04	1,94E-03
cg13178597	RGS17	0,11	0,16	7,55E-04	1,94E-03
cg06072662	RGS17	0,18	0,16	7,55E-04	1,94E-03
cg07811644	NRSN1	-0,15	-0,01	7,71E-04	1,97E-03
cg03284310	HMX3	-0,14	-0,07	9,90E-04	2,51E-03
cg21657577	ZNF578	-0,22	0,24	1,10E-03	2,74E-03
cg12665460	ZNF578	-0,20	0,24	1,10E-03	2,74E-03
cg11909748	ZNF578	-0,15	0,24	1,10E-03	2,74E-03
cg14582763	ZNF578	-0,20	0,24	1,10E-03	2,74E-03
cg13461241	ZNF578	-0,21	0,24	1,10E-03	2,74E-03
cg21553182	ZNF578	-0,18	0,24	1,10E-03	2,74E-03
cg25763393	ZNF578	-0,22	0,24	1,10E-03	2,74E-03
cg15577595	ZNF804A	-0,26	-0,37	2,00E-03	4,94E-03
cg03036557	GPC5	-0,19	0,16	2,07E-03	5,09E-03
cg12678562	GPC5	-0,17	0,16	2,07E-03	5,09E-03
cg10548038	GPC5	-0,18	0,16	2,07E-03	5,09E-03
cg06186301	GPC5	-0,14	0,16	2,07E-03	5,09E-03
cg00302983	SACS	-0,17	0,20	2,09E-03	5,09E-03
cg18836366	SACS	-0,18	0,20	2,09E-03	5,09E-03
cg07175985	SACS	-0,20	0,20	2,09E-03	5,09E-03

cg03734874	TMEM179	-0,18	0,17	3,29E-03	7,98E-03
cg12459037	CNTN4	-0,13	0,44	4,42E-03	1,06E-02
cg02748354	EIF3A	0,14	-0,10	4,69E-03	1,12E-02
cg11237426	NPHS2	-0,23	-0,12	5,00E-03	1,18E-02
cg19284211	INSM1	-0,23	-0,56	5,03E-03	1,19E-02
cg03498096	INSM1	-0,17	-0,56	5,03E-03	1,19E-02
cg06038655	KLF14	-0,20	0,10	6,88E-03	1,61E-02
cg08523865	CNTNAP5	-0,19	-0,16	7,77E-03	1,80E-02
cg01308258	CNTNAP5	-0,15	-0,16	7,77E-03	1,80E-02
cg17321954	STXBP5L	-0,18	-0,45	8,48E-03	1,94E-02
cg07806886	STXBP5L	-0,19	-0,45	8,48E-03	1,94E-02
cg03602730	STXBP5L	-0,14	-0,45	8,48E-03	1,94E-02
cg01855313	STXBP5L	-0,19	-0,45	8,48E-03	1,94E-02
cg07833019	EGFLAM	-0,15	0,25	1,07E-02	2,42E-02
cg00984146	EGFLAM	-0,20	0,25	1,07E-02	2,42E-02
cg24653263	EGFLAM	-0,23	0,25	1,07E-02	2,42E-02
cg06000994	ZNF536	-0,31	0,07	1,50E-02	3,29E-02
cg23331421	ZNF536	-0,24	0,07	1,50E-02	3,29E-02
cg08977371	CDH13	-0,19	0,21	1,86E-02	4,04E-02
cg09825093	CDH13	-0,16	0,21	1,86E-02	4,04E-02
cg08747377	CDH13	-0,27	0,21	1,86E-02	4,04E-02
cg05374412	CDH13	-0,29	0,21	1,86E-02	4,04E-02
cg00806490	CDH13	-0,16	0,21	1,86E-02	4,04E-02
cg06680481	C20orf56	-0,19	0,00	2,18E-02	4,68E-02
cg08582485	C20orf56	-0,23	0,00	2,18E-02	4,68E-02
cg05325193	C20orf56	-0,20	0,00	2,18E-02	4,68E-02
cg06226567	C20orf56	-0,17	0,00	2,18E-02	4,68E-02
cg18299578	FOXG1	-0,26	-0,36	2,72E-02	5,78E-02
cg02681442	FOXG1	-0,24	-0,36	2,72E-02	5,78E-02
cg19553563	TRDN	0,15	-0,43	2,94E-02	6,22E-02
cg16726875	TRDN	0,18	-0,43	2,94E-02	6,22E-02
cg14462830	TRDN	0,15	-0,43	2,94E-02	6,22E-02
cg17889149	TRDN	0,18	-0,43	2,94E-02	6,22E-02
cg02637078	SYT5	-0,19	0,40	3,77E-02	7,82E-02
cg14047019	SYT5	-0,12	0,40	3,77E-02	7,82E-02
cg11960711	ITGB1BP3	-0,12	-0,07	4,29E-02	8,86E-02
cg27601582	ITGB1BP3	-0,20	-0,07	4,29E-02	8,86E-02
cg12208256	CSMD1	-0,25	0,73	4,48E-02	9,23E-02
cg06484232	OR52E4	0,18	-0,07	5,49E-02	1,12E-01
cg27363327	TTBK1	-0,29	-0,32	1,00E-01	2,01E-01
cg09940360	TTBK1	-0,26	-0,32	1,00E-01	2,01E-01
cg07016914	CARTPT	-0,17	0,24	1,07E-01	2,13E-01
cg08743812	CARTPT	-0,19	0,24	1,07E-01	2,13E-01
cg26146287	CARTPT	-0,16	0,24	1,07E-01	2,13E-01
cg02762440	CARTPT	-0,19	0,24	1,07E-01	2,13E-01

cg01187920	CARTPT	-0,25	0,24	1,07E-01	2,13E-01
cg04251750	CARTPT	-0,26	0,24	1,07E-01	2,13E-01
cg07935012	DCC	-0,11	-0,17	1,42E-01	2,80E-01
cg26073933	C1orf61	-0,11	-0,24	2,03E-01	3,91E-01
cg09938227	C1orf61	-0,28	-0,24	2,03E-01	3,91E-01
cg15270145	C1orf61	-0,16	-0,24	2,03E-01	3,91E-01
cg23989821	C14orf39	-0,20	-0,19	2,57E-01	4,89E-01
cg18240143	C14orf39	-0,13	-0,19	2,57E-01	4,89E-01
cg02736505	FAM150B	-0,29	0,50	3,21E-01	6,08E-01
cg10269365	CCDC140	-0,20	-0,01	3,27E-01	6,16E-01
cg27164663	LINGO2	-0,17	0,13	3,31E-01	6,22E-01
cg12658269	LINGO2	-0,19	0,13	3,31E-01	6,22E-01
cg05279757	LINGO2	-0,25	0,13	3,31E-01	6,22E-01
cg26128092	WDR8	-0,25	0,11	3,56E-01	6,67E-01
cg15923139	SORBS2	0,16	0,28	3,83E-01	7,11E-01
cg09260773	TBX15	-0,13	0,18	5,21E-01	9,59E-01
cg19795338	ATP13A4	0,14	0,30	9,53E-01	1,00E+00
cg01250864	IFNGR1	0,14	-0,11	7,10E-01	1,00E+00
cg18041719	SEMA3D	0,13	0,21	9,99E-01	1,00E+00
cg15522953	ZMIZ1	0,16	0,02	9,97E-01	1,00E+00
cg21108805	WIF1	0,12	0,15	1,00E+00	1,00E+00
cg12221621	SPSB1	-0,12	-0,02	1,00E+00	1,00E+00
cg05764238	ARHGEF19	-0,10	0,09	9,57E-01	1,00E+00
cg10595388	SYT6	-0,17	0,04	8,31E-01	1,00E+00
cg01567634	SYT6	-0,18	0,04	8,31E-01	1,00E+00
cg03514545	SYT6	-0,17	0,04	8,31E-01	1,00E+00
cg08883066	SYT6	-0,11	0,04	8,31E-01	1,00E+00
cg03606772	CRCT1	-0,24	-0,16	9,99E-01	1,00E+00
cg00302521	CRCT1	-0,14	-0,16	9,99E-01	1,00E+00
cg09873164	CRCT1	-0,32	-0,16	9,99E-01	1,00E+00
cg03352287	CRCT1	-0,26	-0,16	9,99E-01	1,00E+00
cg01864564	CRCT1	-0,21	-0,16	9,99E-01	1,00E+00
cg19272720	PLEKHA6	0,18	-0,03	1,00E+00	1,00E+00
cg20902122	DISP1	0,13	0,14	8,79E-01	1,00E+00
cg23719318	GDF7	-0,38	0,00	5,67E-01	1,00E+00
cg18552861	GDF7	-0,23	0,00	5,67E-01	1,00E+00
cg05899618	GDF7	-0,14	0,00	5,67E-01	1,00E+00
cg06167664	DPYSL5	-0,24	-0,40	9,96E-01	1,00E+00
cg14483208	DPYSL5	-0,17	-0,40	9,96E-01	1,00E+00
cg10633958	SLC8A1	-0,11	-0,03	1,00E+00	1,00E+00
cg15523958	PLEKHH2	-0,17	0,07	9,95E-01	1,00E+00
cg00778995	POU3F3	-0,27	-0,13	9,81E-01	1,00E+00
cg18524739	POU3F3	-0,18	-0,13	9,81E-01	1,00E+00
cg02583418	POU3F3	-0,25	-0,13	9,81E-01	1,00E+00
cg24925163	SFT2D3	0,11	0,00	1,00E+00	1,00E+00

cg26660002	GALNT13	-0,24	-0,02	1,00E+00	1,00E+00
cg14630357	GALNT13	-0,20	-0,02	1,00E+00	1,00E+00
cg22373593	GALNT13	0,20	-0,02	1,00E+00	1,00E+00
cg23566791	GALNT13	0,19	-0,02	1,00E+00	1,00E+00
cg08813062	SP9	-0,25	-0,02	1,00E+00	1,00E+00
cg14459130	SP9	-0,20	-0,02	1,00E+00	1,00E+00
cg17964510	SP9	-0,24	-0,02	1,00E+00	1,00E+00
cg11528849	NEUROD1	-0,23	-0,14	1,00E+00	1,00E+00
cg16640855	NEUROD1	-0,22	-0,14	1,00E+00	1,00E+00
cg01789478	NEUROD1	-0,15	-0,14	1,00E+00	1,00E+00
cg22359606	NEUROD1	-0,21	-0,14	1,00E+00	1,00E+00
cg06963053	NEUROD1	-0,17	-0,14	1,00E+00	1,00E+00
cg19711579	NEUROD1	-0,14	-0,14	1,00E+00	1,00E+00
cg09942248	NEUROD1	-0,18	-0,14	1,00E+00	1,00E+00
cg06008912	TMEFF2	-0,27	-0,02	9,99E-01	1,00E+00
cg02170986	RFTN2	-0,10	0,10	9,78E-01	1,00E+00
cg01812328	ACADL	0,14	-0,18	1,00E+00	1,00E+00
cg19047707	ECEL1	-0,20	-0,10	1,00E+00	1,00E+00
cg01714811	EOMES	-0,12	-0,18	1,00E+00	1,00E+00
cg02694810	EOMES	-0,18	-0,18	1,00E+00	1,00E+00
cg07287167	ROBO1	-0,18	-0,01	1,00E+00	1,00E+00
cg07526135	STAG1	0,18	-0,13	8,47E-01	1,00E+00
cg26807939	SOX14	-0,18	0,06	1,00E+00	1,00E+00
cg16428251	SOX14	-0,24	0,06	1,00E+00	1,00E+00
cg04228468	SLITRK3	-0,19	0,02	1,00E+00	1,00E+00
cg18447727	SLITRK3	-0,16	0,02	1,00E+00	1,00E+00
cg13619915	SLITRK3	-0,20	0,02	1,00E+00	1,00E+00
cg14717170	SLITRK3	-0,23	0,02	1,00E+00	1,00E+00
cg27363829	SLITRK3	-0,29	0,02	1,00E+00	1,00E+00
cg09269007	SLITRK3	-0,27	0,02	1,00E+00	1,00E+00
cg14502484	SLC6A3	-0,29	-0,23	9,61E-01	1,00E+00
cg17818996	CDH12	0,16	0,12	9,99E-01	1,00E+00
cg00958768	CDH12	0,15	0,12	9,99E-01	1,00E+00
cg14505704	CDH6	-0,15	0,05	9,99E-01	1,00E+00
cg12238343	RXFP3	-0,18	0,03	1,00E+00	1,00E+00
cg00516449	RXFP3	-0,16	0,03	1,00E+00	1,00E+00
cg20963030	ATP10B	0,15	0,09	9,98E-01	1,00E+00
cg05157516	HRH2	-0,27	-0,11	9,85E-01	1,00E+00
cg06847624	PFN3	-0,21	0,01	1,00E+00	1,00E+00
cg00642460	PFN3	-0,18	0,01	1,00E+00	1,00E+00
cg14859460	GRM6	-0,24	0,04	1,00E+00	1,00E+00
cg18417237	LRFN2	-0,15	-0,26	9,85E-01	1,00E+00
cg13290564	HCRTR2	0,14	0,09	9,97E-01	1,00E+00
cg04703174	KHDRBS2	-0,12	-0,02	8,20E-01	1,00E+00
cg11315153	KHDRBS2	-0,16	-0,02	8,20E-01	1,00E+00

cg14224762	RIMS1	-0,15	0,00	1,00E+00	1,00E+00
cg12103152	HTR1B	-0,19	0,02	1,00E+00	1,00E+00
cg23424273	HTR1B	-0,20	0,02	1,00E+00	1,00E+00
cg08614481	HTR1B	-0,24	0,02	1,00E+00	1,00E+00
cg12215457	HTR1B	-0,19	0,02	1,00E+00	1,00E+00
cg13077519	HTR1B	-0,24	0,02	1,00E+00	1,00E+00
cg00183186	HTR1B	-0,24	0,02	1,00E+00	1,00E+00
cg13315970	NT5E	-0,23	-0,01	1,00E+00	1,00E+00
cg16387913	NT5E	0,17	-0,01	1,00E+00	1,00E+00
cg01489686	FUT9	-0,16	-0,44	6,14E-01	1,00E+00
cg20790058	FUT9	-0,19	-0,44	6,14E-01	1,00E+00
cg01815538	PRDM13	-0,16	-0,30	1,00E+00	1,00E+00
cg02400433	PRDM13	-0,20	-0,30	1,00E+00	1,00E+00
cg12227907	PRDM13	-0,18	-0,30	1,00E+00	1,00E+00
cg04974062	PRDM13	-0,19	-0,30	1,00E+00	1,00E+00
cg19223299	PRDM13	-0,23	-0,30	1,00E+00	1,00E+00
cg27125972	PRDM13	-0,12	-0,30	1,00E+00	1,00E+00
cg21239311	NR2E1	-0,26	-0,11	1,00E+00	1,00E+00
cg05135644	NR2E1	-0,21	-0,11	1,00E+00	1,00E+00
cg04407470	NR2E1	-0,34	-0,11	1,00E+00	1,00E+00
cg13776285	NR2E1	-0,28	-0,11	1,00E+00	1,00E+00
cg06103642	NR2E1	-0,20	-0,11	1,00E+00	1,00E+00
cg08929002	NR2E1	-0,16	-0,11	1,00E+00	1,00E+00
cg02149708	T	-0,20	-0,12	9,99E-01	1,00E+00
cg17188046	T	-0,23	-0,12	9,99E-01	1,00E+00
cg19675288	T	-0,22	-0,12	9,99E-01	1,00E+00
cg06463958	T	-0,19	-0,12	9,99E-01	1,00E+00
cg20870512	UNCX	-0,19	-0,01	9,98E-01	1,00E+00
cg14658067	UNCX	-0,15	-0,01	9,98E-01	1,00E+00
cg01202666	TWIST1	-0,20	-0,05	9,97E-01	1,00E+00
cg22498251	TWIST1	-0,16	-0,05	9,97E-01	1,00E+00
cg11124364	TMEM196	-0,13	-0,04	9,81E-01	1,00E+00
cg13298841	HECW1	-0,19	-0,16	1,00E+00	1,00E+00
cg08652532	HECW1	-0,24	-0,16	1,00E+00	1,00E+00
cg13027493	HECW1	-0,13	-0,16	1,00E+00	1,00E+00
cg25717239	HECW1	-0,17	-0,16	1,00E+00	1,00E+00
cg00974941	C7orf57	-0,19	-0,27	9,85E-01	1,00E+00
cg19919208	MAGI2	0,16	-0,07	1,00E+00	1,00E+00
cg11655410	MAGI2	0,12	-0,07	1,00E+00	1,00E+00
cg16111259	MAGI2	0,18	-0,07	1,00E+00	1,00E+00
cg02523844	MAGI2	-0,21	-0,07	1,00E+00	1,00E+00
cg24021956	MAGI2	-0,22	-0,07	1,00E+00	1,00E+00
cg10168361	GNAT3	0,13	-0,21	1,00E+00	1,00E+00
cg24734879	SEMA3D	0,13	0,21	9,99E-01	1,00E+00
cg01182980	SEMA3D	0,17	0,21	9,99E-01	1,00E+00

cg24277791	LHFPL3	-0,18	0,00	1,00E+00	1,00E+00
cg00903099	HTR5A	-0,19	-0,01	1,00E+00	1,00E+00
cg00595237	GATA4	-0,22	-0,03	9,86E-01	1,00E+00
cg27100236	GATA4	-0,18	-0,03	9,86E-01	1,00E+00
cg01509237	NKAIN3	-0,13	-0,03	1,00E+00	1,00E+00
cg07719492	PRDM14	-0,16	0,14	1,00E+00	1,00E+00
cg01610488	TRPA1	-0,16	0,19	1,00E+00	1,00E+00
cg20890210	KCNB2	-0,22	-0,38	8,84E-01	1,00E+00
cg19995049	RPL30	0,16	0,04	1,00E+00	1,00E+00
cg24627900	HPYR1	0,17	0,03	9,87E-01	1,00E+00
cg08326794	BNC2	0,16	-0,11	1,00E+00	1,00E+00
cg08826741	BNC2	0,16	-0,11	1,00E+00	1,00E+00
cg05674150	BNC2	-0,11	-0,11	1,00E+00	1,00E+00
cg17398595	SH3GL2	-0,18	-0,11	1,00E+00	1,00E+00
cg25440961	SH3GL2	-0,19	-0,11	1,00E+00	1,00E+00
cg11258943	FOXE1	-0,26	-0,10	7,06E-01	1,00E+00
cg13724160	GRIN3A	-0,14	-0,14	9,24E-01	1,00E+00
cg05868019	OR13C8	0,17	0,01	9,45E-01	1,00E+00
cg14080475	AKNA	-0,14	-0,02	1,00E+00	1,00E+00
cg11970741	TNC	0,11	0,30	5,98E-01	1,00E+00
cg25042430	KIAA1217	-0,20	-0,08	1,00E+00	1,00E+00
cg18673954	INA	-0,24	-0,52	6,24E-01	1,00E+00
cg23642747	INA	-0,27	-0,52	6,24E-01	1,00E+00
cg21384402	INA	-0,14	-0,52	6,24E-01	1,00E+00
cg24680586	INA	-0,25	-0,52	6,24E-01	1,00E+00
cg04142605	INA	-0,17	-0,52	6,24E-01	1,00E+00
cg13654588	PRLHR	-0,19	-0,03	7,04E-01	1,00E+00
cg12614318	PRLHR	-0,14	-0,03	7,04E-01	1,00E+00
cg02531437	PRLHR	-0,16	-0,03	7,04E-01	1,00E+00
cg19403534	PRLHR	-0,19	-0,03	7,04E-01	1,00E+00
cg14526953	GPR123	-0,23	-0,05	9,88E-01	1,00E+00
cg01178971	KCNQ1	-0,16	0,01	9,90E-01	1,00E+00
cg23178580	OR2AG1	0,17	0,00	1,00E+00	1,00E+00
cg05393484	OR6A2	0,14	-0,02	9,97E-01	1,00E+00
cg10157954	ZNF215	0,15	-0,11	9,98E-01	1,00E+00
cg04270835	SLC17A6	-0,13	-0,12	7,60E-01	1,00E+00
cg22371972	SLC17A6	-0,14	-0,12	7,60E-01	1,00E+00
cg03168749	OR8B12	0,12	0,02	9,99E-01	1,00E+00
cg12859211	ROBO3	-0,19	0,26	1,00E+00	1,00E+00
cg09251429	ROBO3	-0,19	0,26	1,00E+00	1,00E+00
cg01839688	ROBO3	-0,11	0,26	1,00E+00	1,00E+00
cg08833670	ROBO3	-0,18	0,26	1,00E+00	1,00E+00
cg11701621	WNT1	-0,22	0,03	5,48E-01	1,00E+00
cg16112157	KRT6A	0,15	-0,40	9,99E-01	1,00E+00
cg21383810	WIF1	-0,19	0,15	1,00E+00	1,00E+00

cg24719984	PPFIA2	-0,15	-0,23	9,55E-01	1,00E+00
cg24214152	ALX1	-0,26	-0,36	5,75E-01	1,00E+00
cg24083469	ALX1	-0,22	-0,36	5,75E-01	1,00E+00
cg14116122	ALX1	-0,22	-0,36	5,75E-01	1,00E+00
cg07780095	ALX1	-0,25	-0,36	5,75E-01	1,00E+00
cg06552356	ALX1	-0,19	-0,36	5,75E-01	1,00E+00
cg05677041	SRRM4	-0,17	0,06	9,77E-01	1,00E+00
cg02345991	SRRM4	-0,26	0,06	9,77E-01	1,00E+00
cg14843800	SRRM4	-0,20	0,06	9,77E-01	1,00E+00
cg13939792	SIX6	-0,22	-0,07	1,00E+00	1,00E+00
cg19456540	SIX6	-0,28	-0,07	1,00E+00	1,00E+00
cg14511698	SIX6	-0,17	-0,07	1,00E+00	1,00E+00
cg07862488	SIX6	-0,18	-0,07	1,00E+00	1,00E+00
cg00859478	SIX6	-0,20	-0,07	1,00E+00	1,00E+00
cg26517663	FOXB1	-0,19	0,02	1,00E+00	1,00E+00
cg07928083	FOXB1	-0,19	0,02	1,00E+00	1,00E+00
cg07060006	FOXB1	-0,16	0,02	1,00E+00	1,00E+00
cg20987924	FOXB1	-0,30	0,02	1,00E+00	1,00E+00
cg03059131	FOXB1	-0,32	0,02	1,00E+00	1,00E+00
cg03461851	C2CD4B	0,15	-0,18	1,00E+00	1,00E+00
cg01744019	HBM	-0,16	0,05	9,97E-01	1,00E+00
cg09854626	SOX8	-0,21	0,31	8,81E-01	1,00E+00
cg06490869	CRYM	-0,15	-0,23	1,00E+00	1,00E+00
cg20823471	STX1B	-0,19	0,20	5,57E-01	1,00E+00
cg04180299	RLTPR	-0,25	0,36	5,95E-01	1,00E+00
cg21309147	STAC2	-0,14	0,11	1,00E+00	1,00E+00
cg08504407	HRNBP3	-0,14	0,09	7,24E-01	1,00E+00
cg22105145	LOC642597	-0,17	0,05	8,83E-01	1,00E+00
cg00247557	LOC642597	-0,16	0,05	8,83E-01	1,00E+00
cg23234640	LOC642597	-0,12	0,05	8,83E-01	1,00E+00
cg02664349	FAM38B	-0,22	-0,16	1,00E+00	1,00E+00
cg04696345	ZBTB7C	-0,13	0,04	1,00E+00	1,00E+00
cg05928342	ZNF177	-0,20	0,13	1,00E+00	1,00E+00
cg08065231	ZNF177	-0,26	0,13	1,00E+00	1,00E+00
cg17283453	ZNF177	-0,17	0,13	1,00E+00	1,00E+00
cg16055378	VSTM2B	-0,28	0,03	9,90E-01	1,00E+00
cg01464835	VSTM2B	-0,20	0,03	9,90E-01	1,00E+00
cg10743390	VSTM2B	-0,19	0,03	9,90E-01	1,00E+00
cg05056953	DPF1	-0,12	-0,03	1,00E+00	1,00E+00
cg09568464	ZNF582	-0,17	0,06	1,00E+00	1,00E+00
cg22647407	ZNF582	-0,18	0,06	1,00E+00	1,00E+00
cg09861917	FOXA2	-0,23	-0,19	1,00E+00	1,00E+00
cg16963144	FOXA2	-0,24	-0,19	1,00E+00	1,00E+00
cg06672560	CBLN4	-0,15	0,17	1,00E+00	1,00E+00

Supplementary Table S4: Significant CpG probes localized in enhancer regions from hypomethylation global profile in BCVY from metEPICVal study.

Probes	UCSC RefGene Name	Methylation differences (BCVY - BCO)	Gene expression differences (BCVY - BCO)	*p value*	Adjusted *p-value*
cg02950416	BCAN	-0,29	0,79	6,67E-41	9,81E-40
cg20270188	BCAN	-0,22	0,79	6,67E-41	9,81E-40
cg07224636	TMTC1	0,14	0,76	1,56E-28	1,38E-27
cg08729318	TMTC1	-0,12	0,76	1,56E-28	1,38E-27
cg26365957	PTPRS	-0,18	-0,38	4,84E-28	4,21E-27
cg20548934	CADM3	-0,26	1,10	6,46E-28	5,47E-27
cg15891112	CADM3	-0,29	1,10	6,46E-28	5,47E-27
cg03789420	CADM3	-0,17	1,10	6,46E-28	5,47E-27
cg00957516	CADM3	-0,18	1,10	6,46E-28	5,47E-27
cg20724589	HIVEP3	-0,16	0,30	6,77E-25	5,55E-24
cg14839013	HIVEP3	-0,11	0,30	6,77E-25	5,55E-24
cg03938432	BAI3	-0,21	0,47	1,69E-24	1,30E-23
cg10244047	BAI3	-0,13	0,47	1,69E-24	1,30E-23
cg06953874	HOXD9	-0,14	0,43	3,31E-19	2,00E-18
cg10957151	HOXD9	-0,21	0,43	3,31E-19	2,00E-18
cg14142007	HOXD9	-0,23	0,43	3,31E-19	2,00E-18
cg22805974	HOXD9	-0,17	0,43	3,31E-19	2,00E-18
cg14991487	HOXD9	-0,21	0,43	3,31E-19	2,00E-18
cg09578028	HOXD9	-0,24	0,43	3,31E-19	2,00E-18
cg05167251	HOXD9	-0,24	0,43	3,31E-19	2,00E-18
cg03637878	JAM3	-0,20	0,36	4,31E-14	1,84E-13
cg00939226	JAM3	-0,23	0,36	4,31E-14	1,84E-13
cg24899571	JAM3	-0,16	0,36	4,31E-14	1,84E-13
cg02174225	JAM3	-0,17	0,36	4,31E-14	1,84E-13
cg05312305	ZNF655	-0,10	0,36	2,62E-10	9,74E-10
cg11164088	SHANK1	-0,10	0,59	2,80E-10	1,03E-09
cg15805568	SHANK1	-0,11	0,59	2,80E-10	1,03E-09
cg17787134	IGF2BP1	-0,18	-0,51	6,70E-10	2,39E-09
cg27256435	LRCH2	-0,18	0,45	1,17E-08	4,00E-08
cg06321345	FAM5B	-0,13	0,89	3,62E-07	1,11E-06
cg21852439	GRID2	-0,10	0,47	2,05E-06	6,15E-06
cg18629427	GRID2	-0,16	0,47	2,05E-06	6,15E-06
cg20057676	ZIC2	-0,12	-0,83	9,06E-06	2,60E-05
cg24742746	ZIC2	-0,17	-0,83	9,06E-06	2,60E-05
cg17206583	VGF	-0,12	-0,38	9,19E-06	2,63E-05
cg12135573	VGF	-0,17	-0,38	9,19E-06	2,63E-05
cg21657577	ZNF578	-0,22	0,24	1,10E-03	2,74E-03
cg12665460	ZNF578	-0,20	0,24	1,10E-03	2,74E-03
cg11909748	ZNF578	-0,15	0,24	1,10E-03	2,74E-03
cg14582763	ZNF578	-0,20	0,24	1,10E-03	2,74E-03

cg13461241	ZNF578	-0,21	0,24	1,10E-03	2,74E-03
cg21553182	ZNF578	-0,18	0,24	1,10E-03	2,74E-03
cg25763393	ZNF578	-0,22	0,24	1,10E-03	2,74E-03
cg16210670	SNUPN	-0,10	0,08	6,60E-03	1,55E-02
cg07958937	GRB10	-0,10	-0,12	8,06E-03	1,85E-02
cg25848339	GRB10	0,15	-0,12	8,06E-03	1,85E-02
cg03370077	GRB10	0,18	-0,12	8,06E-03	1,85E-02
cg11321337	LRRC7	0,16	0,00	1,32E-02	2,90E-02
cg17163179	LRRC7	0,14	0,00	1,32E-02	2,90E-02
cg08977371	CDH13	-0,19	0,21	1,86E-02	4,04E-02
cg09825093	CDH13	-0,16	0,21	1,86E-02	4,04E-02
cg08747377	CDH13	-0,27	0,21	1,86E-02	4,04E-02
cg05374412	CDH13	-0,29	0,21	1,86E-02	4,04E-02
cg00806490	CDH13	-0,16	0,21	1,86E-02	4,04E-02
cg18299578	FOXG1	-0,26	-0,36	2,72E-02	5,78E-02
cg02681442	FOXG1	-0,24	-0,36	2,72E-02	5,78E-02
cg12204957	IRAK2	0,15	0,22	6,06E-02	1,23E-01
cg17410236	FLRT2	-0,14	0,40	1,73E-01	3,38E-01
cg01711160	FLRT2	-0,23	0,40	1,73E-01	3,38E-01
cg18267374	NEFM	-0,20	0,12	1,82E-01	3,56E-01
cg07552803	NEFM	-0,19	0,12	1,82E-01	3,56E-01
cg20701183	LMO4	-0,13	-0,26	3,57E-01	6,68E-01
cg08603642	ROR1	0,15	0,00	1,00E+00	1,00E+00
cg06167664	DPYSL5	-0,24	-0,40	9,96E-01	1,00E+00
cg14483208	DPYSL5	-0,17	-0,40	9,96E-01	1,00E+00
cg15706621	SPTBN1	-0,12	-0,03	1,00E+00	1,00E+00
cg05276829	SPTBN1	-0,11	-0,03	1,00E+00	1,00E+00
cg00778995	POU3F3	-0,27	-0,13	9,81E-01	1,00E+00
cg18524739	POU3F3	-0,18	-0,13	9,81E-01	1,00E+00
cg02583418	POU3F3	-0,25	-0,13	9,81E-01	1,00E+00
cg08813062	SP9	-0,25	-0,02	1,00E+00	1,00E+00
cg14459130	SP9	-0,20	-0,02	1,00E+00	1,00E+00
cg17964510	SP9	-0,24	-0,02	1,00E+00	1,00E+00
cg20415809	ITGA4	-0,20	-0,11	9,86E-01	1,00E+00
cg06952671	ITGA4	-0,26	-0,11	9,86E-01	1,00E+00
cg25024074	ITGA4	-0,22	-0,11	9,86E-01	1,00E+00
cg16057262	ITGA4	-0,14	-0,11	9,86E-01	1,00E+00
cg06008912	TMEFF2	-0,27	-0,02	9,99E-01	1,00E+00
cg05911659	CPLX2	-0,20	0,01	9,94E-01	1,00E+00
cg23495748	CPLX2	-0,18	0,01	9,94E-01	1,00E+00
cg19885761	CPLX2	-0,22	0,01	9,94E-01	1,00E+00
cg16590189	CPLX2	-0,25	0,01	9,94E-01	1,00E+00
cg26982364	CPLX2	-0,14	0,01	9,94E-01	1,00E+00
cg23409774	CPLX2	-0,21	0,01	9,94E-01	1,00E+00
cg14224762	RIMS1	-0,15	0,00	1,00E+00	1,00E+00

cg21717549	VGLL2	-0,20	0,02	1,00E+00	1,00E+00
cg08983097	VGLL2	-0,26	0,02	1,00E+00	1,00E+00
cg13371788	VGLL2	-0,20	0,02	1,00E+00	1,00E+00
cg20870512	UNCX	-0,19	-0,01	9,98E-01	1,00E+00
cg14658067	UNCX	-0,15	-0,01	9,98E-01	1,00E+00
cg16842053	CARD11	-0,19	0,01	1,00E+00	1,00E+00
cg00595237	GATA4	-0,22	-0,03	9,86E-01	1,00E+00
cg27100236	GATA4	-0,18	-0,03	9,86E-01	1,00E+00
cg01610488	TRPA1	-0,16	0,19	1,00E+00	1,00E+00
cg05191879	C8orf73	-0,17	0,23	9,97E-01	1,00E+00
cg12859211	ROBO3	-0,19	0,26	1,00E+00	1,00E+00
cg09251429	ROBO3	-0,19	0,26	1,00E+00	1,00E+00
cg01839688	ROBO3	-0,11	0,26	1,00E+00	1,00E+00
cg08833670	ROBO3	-0,18	0,26	1,00E+00	1,00E+00
cg09143195	CNTN1	-0,16	-0,11	1,00E+00	1,00E+00
cg15087347	CNTN1	-0,16	-0,11	1,00E+00	1,00E+00
cg19987210	AVPR1A	-0,29	-0,11	1,00E+00	1,00E+00
cg09040797	AVPR1A	-0,25	-0,11	1,00E+00	1,00E+00
cg10862431	AVPR1A	-0,25	-0,11	1,00E+00	1,00E+00
cg27032502	AVPR1A	-0,19	-0,11	1,00E+00	1,00E+00
cg13631391	AVPR1A	-0,21	-0,11	1,00E+00	1,00E+00
cg17771605	LOC283392	-0,21	0,40	9,85E-01	1,00E+00
cg01030121	LOC283392	-0,29	0,40	9,85E-01	1,00E+00
cg09972192	LOC283392	-0,14	0,40	9,85E-01	1,00E+00
cg00580978	LOC283392	-0,16	0,40	9,85E-01	1,00E+00
cg13099890	LOC283392	-0,25	0,40	9,85E-01	1,00E+00
cg19516457	LOC283392	-0,14	0,40	9,85E-01	1,00E+00
cg26517663	FOXB1	-0,19	0,02	1,00E+00	1,00E+00
cg07928083	FOXB1	-0,19	0,02	1,00E+00	1,00E+00
cg07060006	FOXB1	-0,16	0,02	1,00E+00	1,00E+00
cg20987924	FOXB1	-0,30	0,02	1,00E+00	1,00E+00
cg03059131	FOXB1	-0,32	0,02	1,00E+00	1,00E+00
cg04180299	RLTPR	-0,25	0,36	5,95E-01	1,00E+00
cg02701059	HNF1B	-0,22	-0,02	9,98E-01	1,00E+00
cg14054928	CA10	-0,24	-0,16	7,80E-01	1,00E+00
cg14073722	CA10	-0,23	-0,16	7,80E-01	1,00E+00
cg14231749	CA10	-0,13	-0,16	7,80E-01	1,00E+00
cg04946749	MGAT5B	-0,24	0,09	9,99E-01	1,00E+00
cg09568464	ZNF582	-0,17	0,06	1,00E+00	1,00E+00
cg22647407	ZNF582	-0,18	0,06	1,00E+00	1,00E+00
cg10439765	SLC12A5	-0,16	0,07	9,95E-01	1,00E+00

Supplementary Table S5: Significant CpG probes localized in enhancer regions from hypermethylation distinctive signature in BCVY from metEPICVal study.

Probes	UCSC RefGene Name	Methylation differences (BCVY - BCO)	Gene expression differences (BCVY - BCO)	*p value*	Adjusted *p-value*
cg08494221	PURG	-0,10	0,54	1,01E-49	4,57E-48
cg14185504	FIG4	0,13	-0,24	1,53E-13	9,92E-13
cg00670438	PYGL	-0,18	-0,33	5,60E-06	2,06E-05
cg19839026	PYGL	-0,15	-0,33	5,60E-06	2,06E-05
cg07035145	PYGL	-0,17	-0,33	5,60E-06	2,06E-05
cg14483244	HDAC5	-0,10	0,23	1,05E-04	3,39E-04
cg10142997	PCDH9	0,11	0,36	8,54E-04	2,47E-03
cg12204957	IRAK2	0,15	0,23	2,45E-02	6,23E-02
cg17237907	MICAL1	-0,10	0,08	3,78E-01	7,56E-01
cg10807084	AK5	-0,13	0,39	1,00E+00	1,00E+00
cg26440512	ABLIM2	0,10	-0,04	9,71E-01	1,00E+00
cg01708617	RAI14	-0,10	-0,10	9,52E-01	1,00E+00
cg18181545	EDIL3	0,11	-0,49	6,56E-01	1,00E+00
cg10070202	OSBP2	0,10	-0,01	9,38E-01	1,00E+00

Supplementary Table S6: Significant CpG probes localized in TFBS regions from hypermethylation distinctive signature in BCVY from metEPICVal study.

Probes	UCSC RefGene Name	Methylation differences (BCVY - BCO)	Gene expression differences (BCVY - BCO)	*p value*	Adjusted *p-value*
cg26581860	EHF	0,16	-1,18	1,49E-26	1,76E-25
cg04788216	EHF	0,18	-1,18	1,49E-26	1,76E-25
cg09974610	EHF	0,20	-1,18	1,49E-26	1,76E-25
cg22596683	EHF	0,17	-1,18	1,49E-26	1,76E-25
cg20823324	TMED3	0,12	-0,29	2,50E-24	2,72E-23
cg12033458	UNC5C	0,15	0,73	6,45E-20	6,75E-19
cg06571387	HOXD12	-0,11	-0,07	1,12E-19	1,13E-18
cg23167136	CSRP1	0,14	0,35	8,41E-18	7,63E-17
cg19844232	ASAP1	-0,12	-0,27	6,67E-17	5,67E-16
cg06261500	SND1	0,14	-0,12	3,01E-14	2,10E-13
cg06026636	SND1	0,15	-0,12	3,01E-14	2,10E-13
cg14599421	DTNB	0,15	-0,35	1,81E-13	1,14E-12
cg14022913	NEIL3	0,13	-0,62	1,85E-13	1,14E-12
cg27211179	EXD2	0,10	0,14	1,40E-10	7,05E-10
cg05293827	PDE1C	0,14	0,65	3,21E-10	1,59E-09
cg03522150	C11orf49	0,11	0,26	8,75E-10	4,25E-09
cg25029197	KRT7	0,14	-0,50	2,50E-04	7,80E-04
cg05966408	NRG2	0,10	0,61	2,60E-04	8,03E-04
cg15851515	AIM1	0,10	-0,32	4,60E-04	1,36E-03
cg08995655	NUDT19	0,12	-0,22	7,82E-03	2,05E-02
cg12204957	IRAK2	0,15	0,23	2,45E-02	6,23E-02
cg20137977	MCC	0,12	0,25	2,58E-02	6,50E-02
cg19958586	CCRL2	-0,11	-0,16	3,62E-02	8,50E-02
cg22663660	LIPC	0,15	0,36	6,02E-02	1,35E-01
cg00550493	EYS	0,12	-0,27	2,52E-01	5,39E-01
cg03140412	SIGIRR	-0,18	0,21	3,19E-01	6,68E-01
cg14707834	SIGIRR	-0,15	0,21	3,19E-01	6,68E-01
cg13525835	CABLES1	0,12	-0,11	3,67E-01	7,40E-01
cg27451581	KLHDC7A	0,17	-0,21	9,99E-01	1,00E+00
cg24435747	IL28RA	0,13	-0,18	9,81E-01	1,00E+00
cg06957219	DISC1	0,13	-0,04	1,00E+00	1,00E+00
cg07954193	C1D	0,14	-0,05	1,00E+00	1,00E+00
cg05548017	FGD5	0,10	-0,20	9,76E-01	1,00E+00
cg13244293	NGLY1	0,10	-0,06	8,11E-01	1,00E+00
cg10165543	KALRN	-0,12	0,05	1,00E+00	1,00E+00
cg18290075	SGEF	-0,12	0,18	7,42E-01	1,00E+00
cg20045300	FAM114A1	0,14	-0,07	1,00E+00	1,00E+00
cg20956407	NDST1	0,13	-0,10	9,67E-01	1,00E+00
cg23470770	MAP3K5	0,12	0,15	1,00E+00	1,00E+00
cg01250864	IFNGR1	0,14	-0,11	6,99E-01	1,00E+00

cg01687997	C7orf50	0,15	-0,01	1,00E+00	1,00E+00
cg18041719	SEMA3D	0,13	0,29	9,78E-01	1,00E+00
cg19612574	MAPK8	0,11	-0,02	1,00E+00	1,00E+00
cg02820058	PRKG1	0,13	0,13	1,00E+00	1,00E+00
cg20433989	FNDC3A	0,11	-0,05	1,00E+00	1,00E+00
cg10151281	TPM4	0,17	-0,07	1,00E+00	1,00E+00
cg11581475	ZNF428	0,10	0,06	1,00E+00	1,00E+00
cg06058203	MECP2	0,14	0,04	1,00E+00	1,00E+00

Supplementary Table S7. Pathway enrichment results for genes regulated by the CpG probes globally hypomethylated in BCVY.

Reactome Term	P-value	Category	Genes
Neuronal System_Homo sapiens_R-HSA-112316	1.53E-07	Nervious System	GABRB2;SNAP25;GABBR1;KCNC2;CACNA2D1;GABRA4; KCNA1;KCNH8;KCNA6;ADCY8;CPLX1;PTPRD;LRFN5;GNB5;GRIA4
Transmission across Chemical Synapses_Homo sapiens_R-HSA-112315	1.65E-04	Nervious System	GABRB2;SNAP25;GABBR1;CACNA2D1;GABRA4; GNB5;ADCY8;CPLX1;GRIA4
Voltage gated Potassium channels_Homo sapiens_R-HSA-1296072	6.73E-04	Nervious System	KCNC2;KCNH8;KCNA1;KCNA6
G alpha (i) signalling events_Homo sapiens_R-HSA-418594	1.87E-03	Nervious System	RGS4;GRM3;GABBR1;GRM7;GALR1;RGS12;GNB5;ADCY8
Neurotransmitter Receptor Binding And Downstream Transmission In The Postsynaptic Cell_Homo sapiens_R-HSA-112314	2.15E-03	Nervious System	GABRB2;GABBR1;GABRA4;GNB5;ADCY8;GRIA4
SALM protein interactions at the synapse_Homo sapiens_R-HSA-8849932	1.07E-03	Nervious System	PTPRD;LRFN5;GRIA4
GABA receptor activation_Homo sapiens_R-HSA-977443	1.71E-03	Nervious System	GABRB2;GABBR1;GABRA4;ADCY8
Cell-cell junction organization_Homo sapiens_R-HSA-421270	2.50E-03	Cell adhesion	CADM3;PVRL3;F11R;CDH8
Uptake and actions of bacterial toxins_Homo sapiens_R-HSA-5339562	1.96E-03	Bacterial toxins	SNAP25;SV2A;TXNRD1
Potassium Channels_Homo sapiens_R-HSA-1296071	2.33E-03	Nervious System	GABBR1;KCNC2;KCNH8;KCNA1;KCNA6
Nectin/Necl trans heterodimerization_Homo sapiens_R-HSA-420597	1.75E-03	Cell adhesion	CADM3;PVRL3
Adherens junctions interactions_Homo sapiens_R-HSA-418990	2.94E-03	Cell adhesion	CADM3;PVRL3;CDH8
Neurotoxicity of clostridium toxins_Homo sapiens_R-HSA-168799	3.69E-03	Nervious System	SNAP25;SV2A
Glucagon-like Peptide-1 (GLP1) regulates insulin secretion_Homo sapiens_R-HSA-381676	6.96E-03	Regulation of insulin secretion	KCNC2;GNB5;ADCY8
Regulation of insulin secretion_Homo sapiens_R-HSA-422356	6.61E-03	Regulation of insulin secretion	SNAP25;KCNC2;GNB5;ADCY8

Class C/3 (Metabotropic glutamate/pheromone receptors)_Homo sapiens_R-HSA-420499	6.07E-03	G-protein coupled receptor (GPCRs)	GRM3;GABBR1;GRM7
GABA A receptor activation_Homo sapiens_R-HSA-977441	6.27E-03	Nervious System	GABRB2;GABRA4
GPCR ligand binding_Homo sapiens_R-HSA-500792	9.23E-03	G-protein coupled receptor (GPCRs)	GRM3;GABBR1;EDNRB;GRM7;GALR1;AGTR1;GNB5;TAC1;QRFPR;DRD5
G alpha (z) signalling events_Homo sapiens_R-HSA-418597	8.96E-03	Nervious System	RGS4;GNB5;ADCY8
Cell junction organization_Homo sapiens_R-HSA-446728	8.51E-03	Cell adhesion	CADM3;PVRL3;F11R;CDH8
Phase 0 - rapid depolarisation_Homo sapiens_R-HSA-5576892	7.92E-03	Nervious System	CACNG7;CACNA2D1;FGF12
GABA synthesis. release. reuptake and degradation_Homo sapiens_R-HSA-888590	1.33E-02	Nervious System	SNAP25;CPLX1
Serotonin Neurotransmitter Release Cycle_Homo sapiens_R-HSA-181429	1.19E-02	Nervious System	SNAP25;CPLX1
Defective B3GALT6 causes EDSP2 and SEMDJL1_Homo sapiens_R-HSA-4420332	1.33E-02	Extracelular Matrix Organization	BCAN;GPC5
Norepinephrine Neurotransmitter Release Cycle_Homo sapiens_R-HSA-181430	1.19E-02	Nervious System	SNAP25;CPLX1
Acetylcholine Neurotransmitter Release Cycle_Homo sapiens_R-HSA-264642	1.07E-02	Nervious System	SNAP25;CPLX1
Defective B3GAT3 causes JDSSDHD_Homo sapiens_R-HSA-3560801	1.33E-02	Extracelular Matrix Organization	BCAN;GPC5
Defective B4GALT7 causes EDS. progeroid type_Homo sapiens_R-HSA-3560783	1.33E-02	Extracelular Matrix Organization	BCAN;GPC5
Heparan sulfate/heparin (HS-GAG) metabolism_Homo sapiens_R-HSA-1638091	1.39E-02	Extracelular Matrix Organization	BCAN;GPC5;HS3ST4

ECM proteoglycans_Homo sapiens_R-HSA-3000178	1.46E-02	Extracelular Matrix Organization	BCAN;LAMA1;HAPLN1
Integration of energy metabolism_Homo sapiens_R-HSA-163685	1.95E-02	Metabolism	SNAP25;KCNC2;GNB5;ADCY8
Dopamine Neurotransmitter Release Cycle_Homo sapiens_R-HSA-212676	1.91E-02	Nervious System	SNAP25;CPLX1
Glutamate Neurotransmitter Release Cycle_Homo sapiens_R-HSA-210500	2.08E-02	Nervious System	SNAP25;CPLX1
Ligand-gated ion channel transport_Homo sapiens_R-HSA-975298	2.42E-02	Nervious System	GABRB2;GABRA4
Diseases associated with glycosaminoglycan metabolism_Homo sapiens_R-HSA-3560782	2.42E-02	Extracelular Matrix Organization	BCAN;GPC5
Glycosaminoglycan metabolism_Homo sapiens_R-HSA-1630316	2.65E-02	Extracelular Matrix Organization	BCAN;GPC5;HS3ST4;PAPSS2
Phase 1 - inactivation of fast Na+ channels_Homo sapiens_R-HSA-5576894	2.59E-02	Nervious System	CACNG7;CACNA2D1
G alpha (q) signalling events_Homo sapiens_R-HSA-416476	3.32E-02	Nervious System	EDNRB;AGTR1;GNB5;TAC1;QRFPR
Cell-Cell communication_Homo sapiens_R-HSA-1500931	3.41E-02	Cell adhesion	CADM3;PVRL3;F11R;CDH8
Peptide ligand-binding receptors_Homo sapiens_R-HSA-375276	3.45E-02	Cell adhesion	EDNRB;GALR1;AGTR1;TAC1;QRFPR
Amino acid transport across the plasma membrane_Homo sapiens_R-HSA-352230	3.35E-02	Nervious System	SLC6A15;SLC7A3
HS-GAG biosynthesis_Homo sapiens_R-HSA-2022928	3.35E-02	Extracelular Matrix Organization	GPC5;HS3ST4
Extracellular matrix organization_Homo sapiens_R-HSA-1474244	4.93E-02	Extracelular Matrix Organization	BCAN;LAMA1;ADAMTS18;F11R;HAPLN1;JAM3
Diseases of glycosylation_Homo sapiens_R-HSA-3781865	4.89E-02	Extracelular Matrix Organization	BCAN;ADAMTS18;GPC5
Tachykinin receptors bind tachykinins_Homo sapiens_R-HSA-380095	4.56E-02	Cell adhesion	TAC1

Supplementary Table S8. Pathway enrichment results for genes regulated by CpG probes distinctively hypermethylated in BCVY.

Reactome Term	P-value	Category	Genes
Nef mediated downregulation of MHC class I complex cell surface expression_Homo sapiens_R-HSA-164940	2.10E-04	Immune system related pathways	AP1B1;AP1S3
Nef-mediates down modulation of cell surface receptors by recruiting them to clathrin adapters_Homo sapiens_R-HSA-164938	9.67E-04	Immune system related pathways	AP1B1;AP1S3
The role of Nef in HIV-1 replication and disease pathogenesis_Homo sapiens_R-HSA-164952	1.72E-03	Immune system related pathways	AP1B1;AP1S3
Lysosome Vesicle Biogenesis_Homo sapiens_R-HSA-432720	2.69E-03	Vesicular traffic and biogenesis	AP1B1;AP1S3
NOTCH1 Intracellular Domain Regulates Transcription_Homo sapiens_R-HSA-2122947	4.80E-03	Signaling by NOTCH/NOTCH1	HDAC5;HDAC9
Golgi Associated Vesicle Biogenesis_Homo sapiens_R-HSA-432722	6.29E-03	Vesicular traffic and biogenesis	AP1B1;AP1S3
Signaling by NOTCH1 HD+PEST Domain Mutants in Cancer_Homo sapiens_R-HSA-2894858	6.99E-03	Signaling by NOTCH/NOTCH1	HDAC5;HDAC9
Constitutive Signaling by NOTCH1 HD+PEST Domain Mutants_Homo sapiens_R-HSA-2894862	6.99E-03	Signaling by NOTCH/NOTCH1	HDAC5;HDAC9
Signaling by NOTCH1 in Cancer_Homo sapiens_R-HSA-2644603	6.99E-03	Signaling by NOTCH/NOTCH1	HDAC5;HDAC9
Constitutive Signaling by NOTCH1 PEST Domain Mutants_Homo sapiens_R-HSA-2644606	6.99E-03	Signaling by NOTCH/NOTCH1	HDAC5;HDAC9
Signaling by NOTCH1 PEST Domain Mutants in Cancer_Homo sapiens_R-HSA-2644602	6.99E-03	Signaling by NOTCH/NOTCH1	HDAC5;HDAC9
Clathrin derived vesicle budding_Homo sapiens_R-HSA-421837	1.04E-02	Vesicular traffic and biogenesis	AP1B1;AP1S3
trans-Golgi Network Vesicle Budding_Homo sapiens_R-HSA-199992	1.04E-02	Vesicular traffic and biogenesis	AP1B1;AP1S3
Vitamin B2 (riboflavin) metabolism_Homo sapiens_R-HSA-196843	1.10E-02	Vitamine B2 metabolism	ENPP1
Signaling by NOTCH1_Homo sapiens_R-HSA-1980143	1.10E-02	Signaling by NOTCH/NOTCH1	HDAC5;HDAC9
Transport and synthesis of PAPS_Homo sapiens_R-HSA-174362	1.31E-02	PAPS synthesis	PAPSS2
GDP-fucose biosynthesis_Homo sapiens_R-HSA-6787639	1.31E-02	Sugar biosyntesis	GMDS

Membrane Trafficking_Homo sapiens_R-HSA-199991	1.34E-02	Vesicular traffic and biogenesis	KIF26B;AP1B1;TMED3;AP1S3
COPI-dependent Golgi-to-ER retrograde traffic_Homo sapiens_R-HSA-6811434	1.37E-02	Vesicular traffic and biogenesis	KIF26B;TMED3
MHC class II antigen presentation_Homo sapiens_R-HSA-2132295	2.16E-02	Immune system related pathways	AP1B1;AP1S3
Synthesis of PIPs at the late endosome membrane_Homo sapiens_R-HSA-1660517	2.18E-02	PI metabolism and related pathways	FIG4
Removal of the Flap Intermediate from the C-strand_Homo sapiens_R-HSA-174437	2.18E-02	DNA repair	RPA3
Vesicle-mediated transport_Homo sapiens_R-HSA-5653656	2.26E-02	Vesicular traffic and biogenesis	KIF26B;AP1B1;TMED3;AP1S3
Processive synthesis on the C-strand of the telomere_Homo sapiens_R-HSA-174414	2.39E-02	DNA repair	RPA3
Golgi-to-ER retrograde transport_Homo sapiens_R-HSA-8856688	2.44E-02	Vesicular traffic and biogenesis	KIF26B;TMED3
Signaling by NOTCH_Homo sapiens_R-HSA-157118	2.96E-02	Signaling by NOTCH/NOTCH1	HDAC5;HDAC9
Mismatch repair (MMR) directed by MSH2:MSH6 (MutSalpha)_Homo sapiens_R-HSA-5358565	3.04E-02	DNA repair	RPA3
Mismatch repair (MMR) directed by MSH2:MSH3 (MutSbeta)_Homo sapiens_R-HSA-5358606	3.04E-02	DNA repair	RPA3
Removal of the Flap Intermediate_Homo sapiens_R-HSA-69166	3.04E-02	DNA repair	RPA3
Synthesis of PIPs at the early endosome membrane_Homo sapiens_R-HSA-1660516	3.04E-02	PI metabolism and related pathways	FIG4
Mismatch Repair_Homo sapiens_R-HSA-5358508	3.25E-02	DNA repair	RPA3
Processive synthesis on the lagging strand_Homo sapiens_R-HSA-69183	3.25E-02	DNA repair	RPA3
Host Interactions of HIV factors_Homo sapiens_R-HSA-162909	3.27E-02	Immune system related pathways	AP1B1;AP1S3
Translesion synthesis by REV1_Homo sapiens_R-HSA-110312	3.46E-02	DNA repair	RPA3
Translesion synthesis by POLI_Homo sapiens_R-HSA-5656121	3.68E-02	DNA repair	RPA3
Translesion synthesis by POLK_Homo sapiens_R-HSA-5655862	3.68E-02	DNA repair	RPA3

ABC transporters in lipid homeostasis_Homo sapiens_R-HSA-1369062	3.68E-02	Lipid transport	PEX3
Formation of Senescence-Associated Heterochromatin Foci (SAHF)_Homo sapiens_R-HSA-2559584	3.68E-02	Senescence	UBN1
Synthesis of PIPs at the Golgi membrane_Homo sapiens_R-HSA-1660514	3.89E-02	PI metabolism and related pathways	FIG4
Translesion Synthesis by POLH_Homo sapiens_R-HSA-110320	4.10E-02	DNA repair	RPA3
PCNA-Dependent Long Patch Base Excision Repair_Homo sapiens_R-HSA-5651801	4.10E-02	DNA repair	RPA3
Lagging Strand Synthesis_Homo sapiens_R-HSA-69186	4.31E-02	DNA repair	RPA3
Telomere C-strand (Lagging Strand) Synthesis_Homo sapiens_R-HSA-174417	4.73E-02	DNA repair	RPA3
Resolution of AP sites via the multiple-nucleotide patch replacement pathway_Homo sapiens_R-HSA-110373	4.73E-02	DNA repair	RPA3
Gap-filling DNA repair synthesis and ligation in GG-NER_Homo sapiens_R-HSA-5696397	4.94E-02	DNA repair	RPA3

Supplementary Table S9: Results of qRT-PCR validation analysis.

**Gene**	**BCVY mean**	**BCO mean**	**Mean differences**	***p-value***
**NEIL3**	1.91	0.58	1.33	$1x10^{-4}$*
**PAPSS2**	12.37	3.43	8.94	0.001*
**HOXD9**	6.47	1.27	5.20	0.005*
**HDAC5**	19.4	4.46	14.94	0.010*
**HDAC9**	7.76	3.08	4.68	0.037*
**AP1B1**	5.64	2.89	2.75	0.037*
**PCDH10**	10.82	2.14	8.68	0.047*
**TIGIT**	20.08	4.61	15.47	0.081
**EHF**	0.96	1.76	-0.80	0.248
**CXCL17**	0.88	0.99	-0.11	0.502
**AP1S3**	1.93	3.12	-1.19	0.74
**OTUD3**	1.27	1.2	0.07	0.859
**PEX3**	1.79	1.47	0.32	0.896
