# Supplementary material for: Acceleration in the DNA methylation age in breast cancer tumours from very young women
Source: Sci Rep. 2019 Oct 18;9:14991. doi: 10.1038/s41598-019-51457-6 (PMC6800453; doi:10.1038/s41598-019-51457-6)
Supplement: Supplementary file 2 — Supplementary Figures [file 41598_2019_51457_MOESM2_ESM.pdf]

Acceleration in the DNA methylation age in breast cancer tumours from very young women

Sara S. Oltra[1], Maria Peña-Chilet[1], Kirsty Flower2, María Teresa Martinez[1], Elisa Alonso[3], Octavio Burgues[3], Ana Lluch[1¥], James M. Flanagan[2], and Gloria Ribas[1¥*]

Supplementary figure S1. Diagram of analysis procedure. Diagram include work flow for the statistical analysis performed for metEPICVal samples. Sig and Sig1/2 = significant probes; N = sample number; Probes = total number of probes for each analysis; Diff = methylation differences between BCVY and BCO; FDR = false discovery rate.

Supplementary figure S2. Global hypomethylation BCVY validation in combined and TCGA data sets. Significant probes from global hypomethylation profile in BCVY obtained from metEPICVal samples were selected and validated in combined data (**A**) and TCGA data (**B**). Green dots represent hypomethylation in BCVY and red dots hypermethylation. Dotted line indicates minus logarithm of adjusted *p-value* threshold applied and red lines delimit methylation differences between BCVY and BCO ±0,1.

metEPICVal study workflow

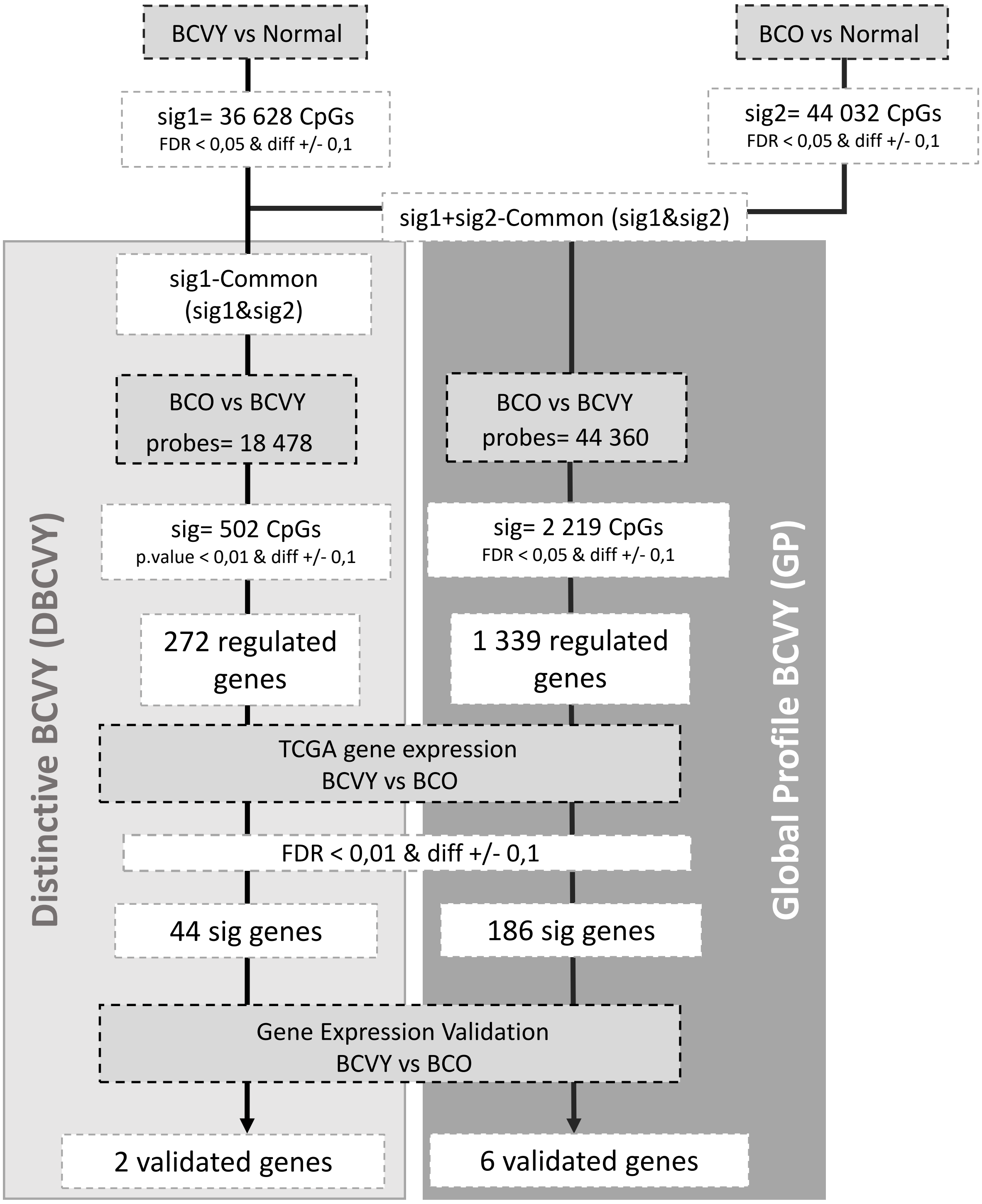

	EPIC study		
	**Methylation**	**TCGA**	**Gene exp Validation**
**BCVY**	N=21	N=27	N=27
**BCO**	N=13	N=720	N=13
**Normal**	N=5	-	-
**Total Probes**	793 483CpGs	1 339 (GP)<br>272 genes (DBCVY)	14 genes

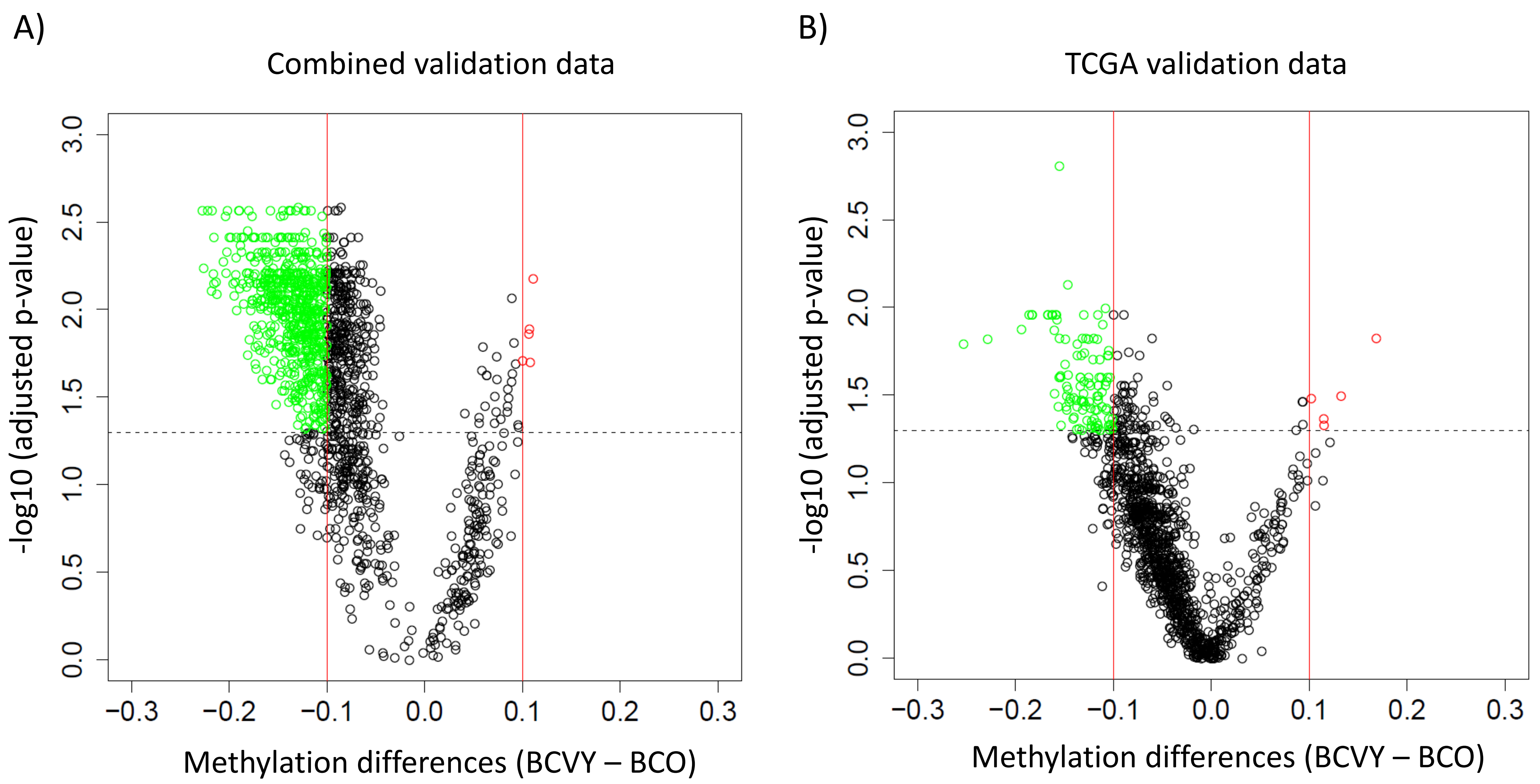
A)
Combined validation data
-log10 (adjusted p-value)
0.0
0.5
1.0
1.5
2.0
2.5
3.0
−0.3
−0.2
−0.1
0.0
0.1
0.2
0.3
Methylation differences (BCVY – BCO)
B)
TCGA validation data
-log10 (adjusted p-value)
0.0
0.5
1.0
1.5
2.0
2.5
3.0
−0.3
−0.2
−0.1
0.0
0.1
0.2
0.3
Methylation differences (BCVY – BCO)
